# Loss of MCT1 mediated lactate uptake causes delayed endplate maturation and intervertebral disc degeneration

**DOI:** 10.1038/s41419-026-08942-4

**Published:** 2026-06-03

**Authors:** Maria Tsingas, Konstantinos Tsingas, Mei Smyers, Wujuan Zhang, Aaron R. Goldman, Eulisa Lawrence, John A. Collins, Makarand V. Risbud

**Affiliations:** 1 Department of Orthopaedic Surgery, Sidney Kimmel Medical College, Thomas Jefferson University, Philadelphia, PA USA; 2 Graduate Program in Cell Biology and Regenerative Medicine, Jefferson College of Life Sciences, Thomas Jefferson University, Philadelphia, PA USA; 3 University of Pennsylvania, Philadelphia, PA USA; 4 Proteomics and Metabolomics Facility, The Wistar Institute, Philadelphia, PA USA; 5 Drexel University, Philadelphia, PA USA

**Keywords:** Cell death, Metabolomics, Cartilage development

## Abstract

During skeletal growth, it is thought that the lactate secreted by the glycolytic nucleus pulposus (NP) cells exits the intervertebral disc into circulation via endplates. Our current studies challenge this long-held notion. Mice with early postnatal, endplate, and annulus fibrosus-specific deletion of lactate importer, MCT1, exhibited disc degeneration characterized by NP cell loss and pronounced endplate structural changes. Using metabolic and transcriptomic approaches, we demonstrate that MCT1 loss inhibits endplate chondrocyte differentiation and that lactate serves both as a crucial TCA metabolite and promotes protein and histone lactylation and gene expression. These findings suggest that during skeletal growth, NP-derived lactate in part supports endplate cartilage differentiation into the vascularized subchondral bone, which, when absent, limits nutrient exchange and availability to the other disc compartments, affecting their homeostasis. This study provides the first in vivo evidence that loss of MCT1 mediated lactate uptake in endplate cells causes delayed maturation and intervertebral disc degeneration.

## Introduction

The intervertebral disc, the largest avascular organ in the body, is inherently hypoxic. Glucose, the primary energy source for the disc [1–3], diffuses through the vasculature of the bony endplates into the adjacent endplate cartilage and then into the nucleus pulposus (NP) and annulus fibrosus (AF) compartments. The central NP compartment is primarily cellular at birth, but it accumulates a proteoglycan-rich matrix by skeletal maturity. In tandem, in the AF compartment, cells secrete a collagen-rich matrix and develop into an organized, concentric, lamellar structure that persists throughout life. The embryonic endplate is a homogenous cartilaginous tissue that differentiates into two morphologically distinct structures: the cartilaginous endplate (CEP), consisting of 1-2 layers of flattened cells retained throughout life, and a transient cartilage consisting of hypertrophic chondrocytes, which transdifferentiate into osteoblasts, giving rise to an ossified vascularized bony endplate by skeletal maturity [4–6]. During this period of skeletal growth, metabolic demands are likely to be elevated, as disc cells synthesize considerable quantities of extracellular matrix (ECM).

Lactate, the product of anaerobic metabolism, effluxes out of the cell via members of the monocarboxylate transporter (MCT) family of proteins, of which MCT4 is a hypoxia-inducible transporter enriched in glycolytic cells [7, 8]. We have demonstrated the importance of MCT4 in lactate clearance in the NP; inhibition results in lactic acidosis and subsequent disc degeneration [9]. Moreover, MCT4-KO mice exhibit dysregulated endplate maturation, characterized by the persistence of hypertrophic cartilage that fails to differentiate into bone [9]. In a recent study, Wang et al. speculated that lactate from the NP compartment is utilized by neighboring AF cells, implying a metabolic coupling between compartments[10]. However, this scenario is paradoxical considering that the AF, like the NP, is avascular and, thus, unlikely to predominantly rely on oxidative metabolism [11]. This raises an interesting possibility that NP-derived lactate could, in part, be utilized as a metabolite by the surrounding endplate cells [9]. Indeed, MCT1, a bi-directional transporter enriched in EP and AF, preferentially imports lactate across the plasma membrane [10, 12–16]. In the disc microenvironment, the steep lactate concentration gradient generated by glycolytic NP cells thermodynamically favors MCT1-mediated lactate import into adjacent AF and EP cells.

Of particular relevance to lactate metabolism in chondrocytes, a recent study showed that as growth plate chondrocytes progress from a proliferative to a hypertrophic state before transdifferentiating into bone, they metabolically rewire from glycolysis to OXPHOS via IGF2 as a bioenergetic switch [17]. Another study suggested that Sox9-mediated glutamine metabolism is essential for growth plate chondrocyte function, including gene expression, biosynthesis, and redox homeostasis [18]. Relatedly, lactate, which is known to promote glutamine metabolism, can stimulate chondrocyte maturation or act as a metabolic fuel on its own [19]. Lactate serves as a metabolic switch in cancer, increasing pyruvate entry into the TCA cycle while also suppressing glycolysis [20]. Further, Hui et al. developed a tissue-level flux model in mice, which showed that circulating glucose-derived lactate feeds the TCA cycle in most tissues except for the brain [21]. Lactate also serves as a signaling molecule promoting histone and non-histone protein lactylation, via the generation of lactyl-CoA groups, which affects gene expression [22]. In recent years, studies have addressed the mechanisms underlying lactylation. For example, lactyl-CoA modification of histones was found to be promoted by SOX9 and YAP/TEAD binding to the enhancer regions of neural crest cells [23], while HBO1 catalyzes lysine lactylation on histone H3K9 to regulate gene expression [24]. Thus, lactate, which was once considered a waste product, is now considered to serve as the “fulcrum of metabolism” [25].

We hypothesized that the increased abundance of lactate, due to elevated glycolysis in NP, supports EP maturation during skeletal growth. To investigate this, we characterized the spinal phenotype of EP and AF-targeted MCT1 conditional knockout mice, *Slc16a1*^*Col2CreERT2*^ (*Mct1*^*cKO*^) [26, 27]. There was prominent disc degeneration in *Mct1*^*cKO*^ mice, characterized by a significant loss of NP cells and an aberrant matrix. Notably, the discs of *Mct1*^*cKO*^ mice exhibited aberrant endplates that lacked a vascularized bony region and displayed persistence of cartilaginous tissue [17, 18, 28]. These findings provide the first evidence for a growth-linked metabolic coupling between the NP cells and EP chondrocytes. In this way, during postnatal growth, glucose, spared by endplate cells, can be utilized by cells of the NP and AF, which, in return, generate lactate essential for endplate metabolism and maturation, contributing to the maintenance of disc health.

## Results

### *Mct1*^*cKO*^ mice show pronounced disc degeneration and striking endplate alterations

**Fig. 1:**
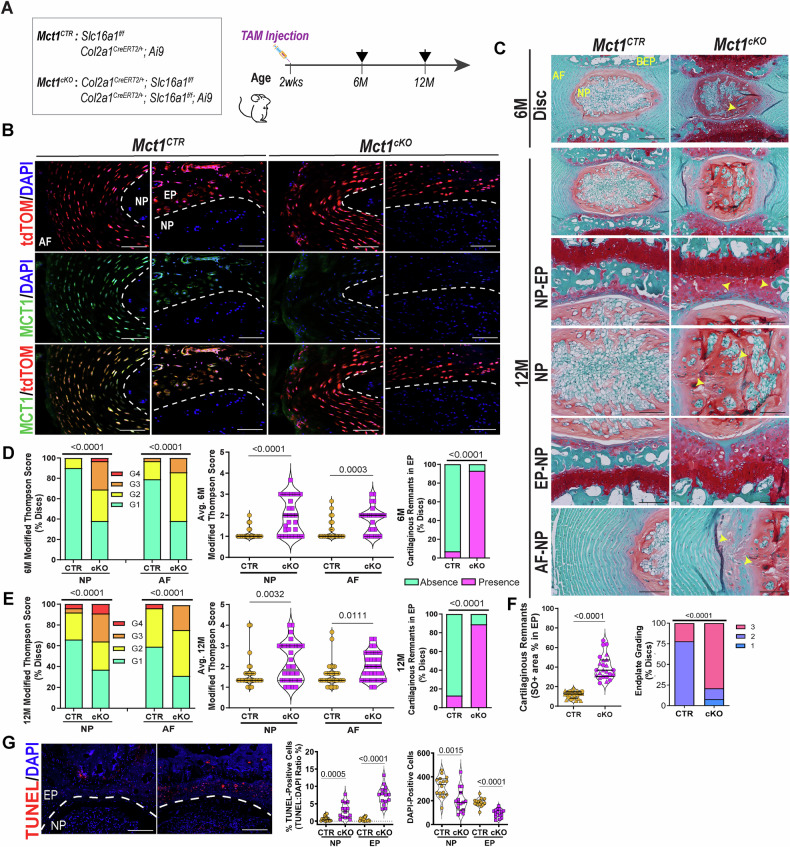
Mct1^cKO^ exhibit pronounced disc degeneration and striking endplate alterations. **A** Overview of the timeline for the generation of Mct1^CTR^ (CTR) and Mct1^cKO^ (cKO) mice. **B** Colocalization of Ai9 (tdTOM + ) reporter marking Cre recombinase activity with MCT1 shows MCT deletion in the AF and EP compartments of cKO mice (scale bar = 50 μm). **C** Safranin O/Fast Green staining of the discs from 6- and 12-month-old Mct1^CTR^ and Mct1^cKO^ mice. Yellow arrowheads indicate degenerative changes. Scale bar: Rows 1 and 2 = 100 μm; Rows 3–6 = 50 μm. Distribution and average modified Thompson grades, and incidence of EP phenotype in **D** 6-month-old and **E** 12-month-old mice. N; 6 M: 9 mice/genotype (5 males, 4 females), 3-4 caudal discs/mouse, n; 12 M = 11-12 mice/genotype (CTR: 6 f, 5 f; cKO: 6 m, 6 f), 2-4 caudal discs/animal were assessed. **F** Area of persisting EP cartilage shown as Safranin O positive area within the EP, and cartilage grading at 12-month, *n* = 11-12 mice/genotype. **G** Representative TUNEL staining images showing apoptotic cells in the NP and EP regions of disc sections and total number of cells in NP and EP compartments (scale bar = 50 μm), *n* = 5 mice/genotype (3 m, 2 f), 3-4 discs/animal. Data represented as violin plots with median and quartiles. Significance for the grading distribution was determined using Fisher’s Exact test, all other comparisons were performed using an unpaired t-test or Mann-Whitney test based on data normality.

To determine the fate of NP-derived lactate and to understand the consequences of restricted lactate uptake in the neighboring tissues, we generated AF- and EP-targeted MCT1 knockout mice. Tamoxifen was injected at 2 weeks postnatally to induce Cre-mediated recombination, and the phenotypes of *Col2a1*^*CreERT2*^*Slc16a1*^*f/f*^ (*Mct1*^*cKO*^) and *Slc16a1*^*f/f*^ (*Mct1*^*CTR*^) mice were assessed up to 12 months of age (Fig. 1A). In a few crosses, we used an Ai9 tdTomato+ reporter to mark Cre-recombinase activity. As expected, tdTOM+ cells were robustly present in the AF and EP in control (*Ai9*^*tdTomato/+*^*Col2a1*^*CreERT2*^) and knockout discs. Immunostaining revealed a significant loss of MCT1 in the AF and EP cells of *Mct1*^*cKO*^ discs, confirming a robust deletion in the targeted compartments (Fig. 1B, S1A). Safranin O/Fast Green staining of caudal discs of 6- and 12-month skeletally mature *Mct1*^*cKO*^ showed pronounced NP degeneration characterized by a significant decrease in NP cells and loss of demarcation between the NP and AF compartments. Notably, *Mct1*^*cKO*^ endplates, as early as 10 weeks of age, displayed pronounced Safranin-O-positive staining in the bony endplate region, indicating the persistence of cartilaginous tissue (Fig. S1B). Modified Thompson Grading of NP and AF compartments of 6- and 12-month-old mice showed higher grades of degeneration, both in distribution and average, along with a strikingly higher number of discs showing the presence of cartilaginous tissue remnants in the EP of *Mct1*^*cKO*^ (Fig. 1C–F). Moreover, in *Mct1*^*cKO*^ mice, cartilaginous tissue occupied a significantly higher area of the EP and showed higher grades of EP degeneration (Fig.1F). Finally, TUNEL staining revealed a higher incidence of cell death in the NP and EP of *Mct1*^*cKO*^ mice (Fig. 1G). In summary, loss of MCT1 in the EP and AF significantly impacted cellular and extracellular constituents of the intervertebral disc. Notably, our study focused on caudal discs [1], which differ in size, mechanical environment, and cell number from lumbar discs [29, 30] and provide better insights into phenotypes linked to disc metabolism under differing anatomical and physiological conditions.

### MCT1 loss inhibits endochondral ossification and formation of the bony EP

**Fig. 2:**
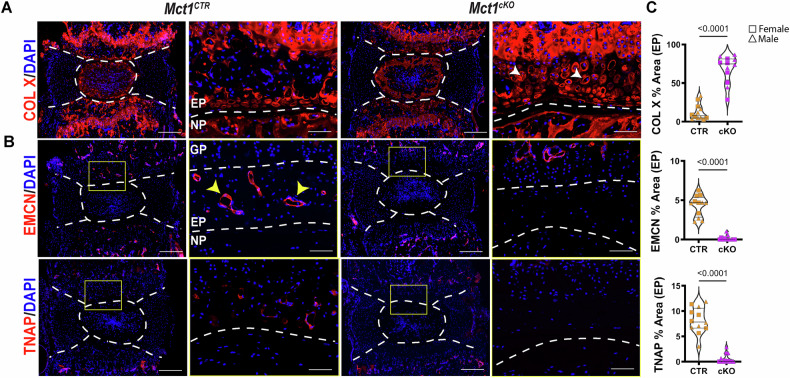
MCT1 loss delays endochondral ossification and formation of the bony EP. **A** Representative images of COL X, **B** EMCN, and TNAP in the endplate region of 12-month-old Mct1^CTR^ and Mct1^cKO^ mice. White arrowheads indicate hypertrophic chondrocytes; yellow arrowheads show blood vessels. Dotted lines indicate boundaries between different tissue compartments within the disc. Scale bar: columns 1, 3 = 200 μm, columns 2, 4, = 50 μm. **C** Corresponding quantification of EP area. Data represented as violin plots with median and quartiles. Significance was determined using an unpaired t-test based on normality. *n* = 4 mice/genotype (2 m, 2 f), 3 discs/animal assessed.

**Fig. 3:**
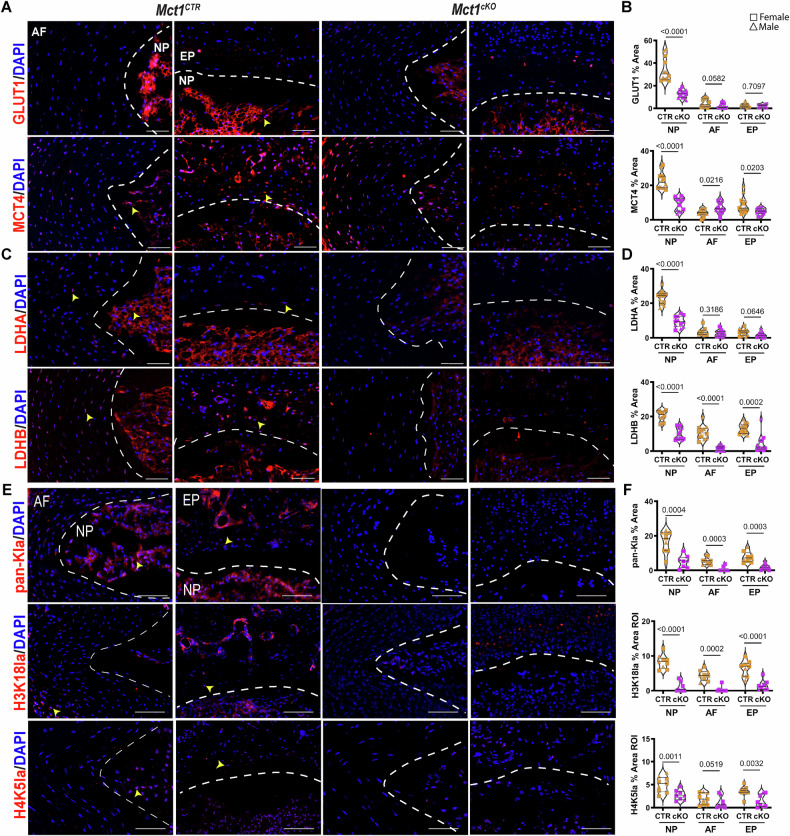
MCT1 loss affects lactate transport and lactylation in the EP of the disc. **A–D** Representative images and corresponding quantification showing GLUT1, MCT4, LDHA, and LDHB in the EP, AF, and NP compartments, scale bar = 50 μm. *n* = 4 mice/genotype (2 m, 2 f), 3 discs/animal assessed. Dotted lines indicate boundaries between different tissue compartments within the disc. **E, F** Representative images and corresponding quantification of 6-month-old discs showing pan-lactylation (pan-Kla), H3K18la and H4K5la, nuclear-specific for lactylation, in the AF-NP and EP, scale bar = 50 μm, *n* = 3 mice/genotype (2 f, 1 m), 2-3 discs/mouse.

To further characterize the phenotype, immunohistological analyses were performed on 12-month-old *Mct1*^*cKO*^ mice. Notably, EP showed persistence of hypertrophic cartilage, as evidenced by robust COL X expression alongside decreased EMCN and TNAP staining, suggesting retention of hypertrophic cartilage, fewer blood vessels, and decreased bone formation (Fig. 2A–C). Consistent with significant NP cell loss and altered morphology, the abundance of NP phenotypic marker GLUT1 was markedly reduced in the *Mct1*^*cKO*^ discs. Furthermore, the staining of the lactate efflux channel MCT4 and enzymes involved in lactate metabolism, LDHA and LDHB, was reduced in the *Mct1*^*cKO*^ discs (Fig. 3A–D). These findings suggest that the inhibition of lactate uptake through MCT1 in EP and AF is sufficient to drive metabolic changes that may contribute to the delayed endochondral ossification observed in the endplate and reduced glycolysis in NP, plausibly reflecting diminished availability of glucose. Noteworthy, we observed abundant pan-Kla staining and nuclear H3K18la and H4K5la localization in EP and NP compartments of 6-month-old *Mct1*^*CTR*^ mice, with a marked reduction in the *Mct1*^*cKO*^ mice (Figs. 3E, F, S2A), suggesting that altered lactate availability affects protein and histone lactylation of disc cells in vivo. Similarly, a reduction in pan-Kla staining was observed in *Mct4* null mice (Fig. S2B).

### *Mct1*^*cKO*^ discs show disorganized ECM and NP fibrosis

*Mct1*^*cKO*^ discs showed significant structural changes, including an increase in thin collagen fibrils, a reduction in intermediate fibril thickness, and notable collagen fibril disorganization in the AF. There was also a marked increase in the incidence and extent of fibrosis of the NP, accompanied by increased COL I expression (Fig. S3A–C). To determine the quality of the collagen matrix, we used a fluorescently labeled peptide (CHP) that binds to denatured collagen [31]. A higher CHP binding in *Mct1*^*cKO*^ disc indicated slight disorganization and an increased abundance of denatured collagen (Fig. S4A). However, there were only subtle changes in aggrecan (ACAN) and chondroitin sulfate staining patterns within NP of *Mct1*^*cKO*^ mice (Fig. S4B).

### MCT1 loss affects endplate mineralization but does not markedly impact vertebral bone

To evaluate whether the endplate phenotype is restricted to the subchondral region or affects the vertebral bone, we performed μCT analysis on 12-month-old mice. Reconstructed three-dimensional scans revealed negligible changes in vertebral bone morphology of *Mct1*^*cKO*^ mice (Fig. S5A). There were no changes in disc height or disc height index (DHI); however, there was a small decrease in vertebral length (Fig. S5B). Cortical and trabecular bone properties were assessed and showed no major differences, except for a significant increase in trabecular and cortical BMD (Fig. S5C). *Mct1*^*cKO*^ mice displayed a significant decrease in endplate BMD, consistent with the histological outcomes, which showed the persistence of cartilage (Fig. S5D).

### MCT1 loss does not affect knee cartilage degradation

To determine whether structural alterations in *Mct1*^*cKO*^ mice were restricted to the vertebral endplate or affected other cartilages, knee osteoarthritis (OA) severity was assessed using μCT and histology of the hindlimbs in 12-month-old mice. μCT analysis of tibial subchondral bone volume fraction (BV/TV), Trabecular Thickness (Tb.Th), Trabecular Separation (Tb.Sp), and Subchondral Bone Plate thickness (SCBP.Th) was conducted on the medial tibial plateaus of *Mct1*^*CTR*^ and *Mct1*^*cKO*^ mice where no changes were observed between genotypes (Fig. S6A, B). Histologically, neither *Mct1*^*CTR*^ nor *Mct1*^*cKO*^ mice developed signs of cartilage degradation, characterized by changes in articular cartilage structure (ACS) (Fig. S6C).

### Spatial transcriptomics reveals altered expression profiles and cell fate in *Mct1*^*cKO*^ discs

**Fig. 4:**
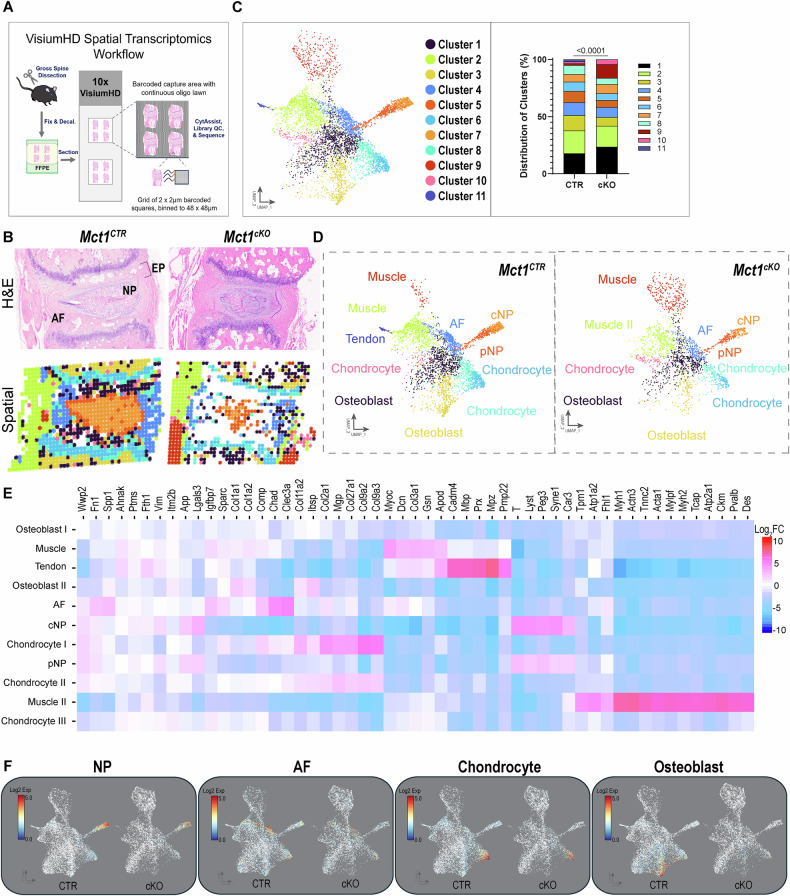
Spatial transcriptomics unveils altered transcriptomic profiles and cell fate in Mct1^cKO^. **A** Spatial transcriptomics workflow using VisiumHD platform for spinal motion segments of 6-month-old Mct1^CTR^ and Mct1^cKO^ mice. **B** H&E-stained images of a control and knockout mouse spinal motion segment (caudal level 7/8) and the corresponding spatial profiles following clustering analysis. **C** UMAP showing 11 distinct cell clusters identified by Seurat package using merged data set from CTR and cKO mice and stacked bar distribution of clusters between genotypes, *n* = 4 mice/genotype; 2 m, 2 f. **D** Cluster annotated UMAP plots of Mct1^CTR^ and Mct1^cKO^ showing distinct cell populations with the disc compartment. **E** Heatmap of expressed markers used for cluster annotation. **F** Feature plots showing spatial expression of combined marker genes in the NP, AF, chondrocyte, and osteoblast populations in CTR and cKO mice.

We utilized spatial transcriptomics to gain insights into gene expression changes in *Mct1*^*cKO*^ discs (Fig. 4A). This approach enabled the capture of positional information at single-cell resolution, including crosstalk between disc compartments, which is otherwise lost in scRNA-sequencing techniques. H&E images, with corresponding spatial maps of the *Mct1*^*CTR*^ and *Mct1*^*cKO*^ motion segments, indicated distinct tissue-specific clustering (Fig. 4B) in the ROI defined between superior and inferior growth plates. Seurat clustering analysis showed 11 distinct cell clusters, with a significant difference in overall distribution of clusters between genotypes (Fig. 4C). Clusters were annotated from a merged dataset based on top marker genes (Fig. 4D–F). The NP was defined by the combined expression of *Krt19*, *T*, *Acan*, and *Krt8*. The AF was annotated based on the expression of *Chad*, *COMP*, *Clec3a*, and *Fn1*. *Col2a1*, *Col10a1*, *Mgp*, *Col11a1*, and *Col9a1* were used to define the chondrocyte cluster. The osteoblast cluster was determined by expression of *Runx2a*, *Col1a1*, *Sp7, Dmp1, Ibsp*. When these clusters were compared, distinct differences were noted between genotypes (Fig. 4F; S7A–D). Furthermore, we observed two distinct NP populations, periNP (pNP) and centriNP (cNP), which clustered distinctly from the other cell types. Chondrocyte, muscle, and osteoblast cell populations comprised multiple clusters labeled as Clusters “I”, “II”, or “III”, whereas AF and tendon cells comprised a single cluster each. *Mct1*^*cKO*^ samples showed negligible transcriptomic differences in the clustering of AF cells, and the lack of a tendon cluster was likely due to sample processing. Interestingly, *Mct1*^*cKO*^ showed differences in the positioning of certain clusters in spatial maps; in particular, osteoblasts were largely absent from the bony endplate region of mutant mice, consistent with the histological observation that the bony endplate was poorly structured (Fig. 4B).

**Fig. 5:**
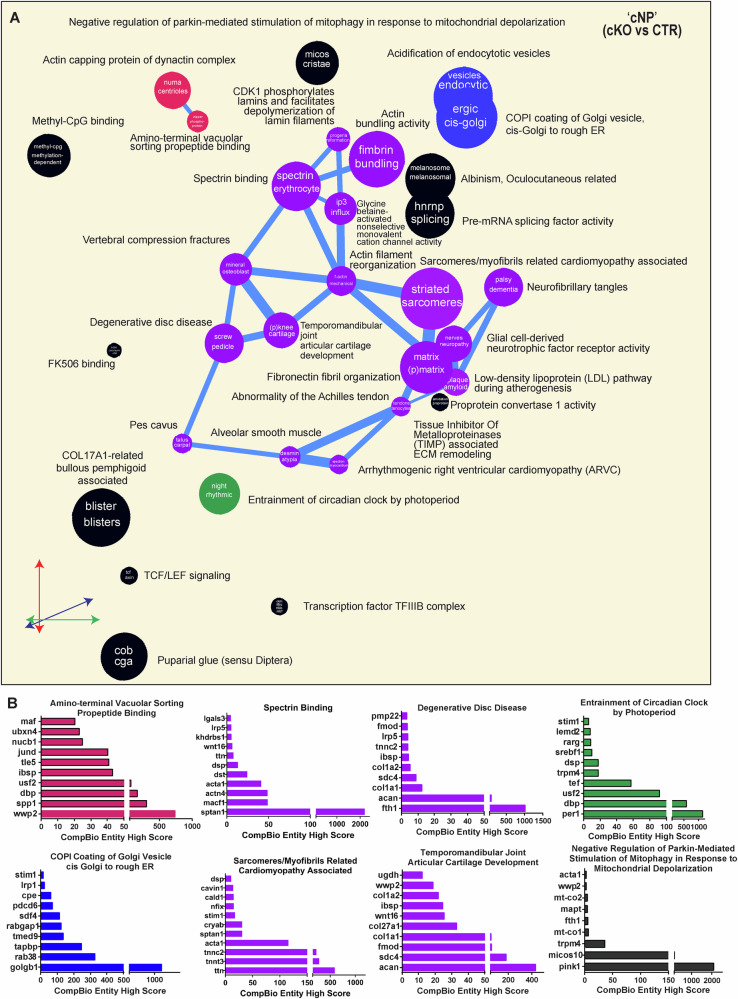
MCT1 deletion dysregulates the transcriptomic signatures associated with the ‘central NP’ cell cluster. **A** Thematic organization of concepts generated by CompBio, derived from literature analysis of differentially expressed genes in the ‘central NP’ cluster of the Mct1^cKO^ mouse discs, reveals key physiological processes affected by impaired lactate uptake. Superclusters of themes associated with these processes include homeostasis and development (purple), actin and protein binding (red), endocytosis and vesicular trafficking (blue), and circadian rhythm regulation (green). **B** Top DEGs enriched in key themes are displayed as entities based on their CompBio entity high score, 4 mice/genotype; 2 m, 2 f.

To examine the genotype-based differences between cell clusters with the same annotation, significantly differentially expressed genes (DEGs) were identified (FDR < 0.05). Notably, the ‘cNP’ cluster had 108 downregulated and 20 upregulated DEGs (Fig. S8A). Pathway analysis of the cluster DEGs was carried out using the AI CompBio tool (PercayAI Inc., St. Louis, MO) to explore thematic associations between these genes (Fig. 5A, B). A ball-and-stick plot of DEGs from the ‘cNP’ cluster revealed a prominent thematic supercluster, associated with muscle structure and cytoskeletal processes. Key themes within this supercluster included *Degenerative Disc Disease*, *Temporomandibular Joint Articular Cartilage Development*, *Actin Bundling Activity*, and *Fibronectin Fibril Organization*. The top genes contributing to these themes include *Acan*, *Fth1*, *Sdc4*, and *Ibsp*, which highlight significant changes in disc homeostasis. Another distinct supercluster focused on actin and protein binding, featuring themes such as: *Amino-terminal Vacuolar Sorting Propeptide Binding and Actin Capping Protein of Dynactin Complex*. Additionally, a smaller supercluster related to endocytosis and vesicular trafficking encompassed themes like *Acidification of Endocytotic Vesicles and COPI Coating of Golgi Vesicle cis Golgi to rough ER*, with key genes including *Golgb1*, *Cavin1*, and *Cltb*. Overall, these findings indicate that the cNP of *Mct1*^*cKO*^ mice exhibits significant alterations in the cellular phenotype, accompanied by degenerative changes. Gene set enrichment analysis (GSEA) was conducted on DEGs defined by *q*-values of 0.25 and 0.05. In cNP cells of *Mct1*^*cKO*^ mice, GSEA showed upregulation in pathways related to muscle: ‘actin-myosin and muscle filament sliding’ and ‘muscle contraction’, supporting altered cell phenotype and tissue fibrosis (Fig. S8B, C).

We analyzed the differences in the ‘Chondrocyte I’ cluster between genotypes and found 39 downregulated and 3 upregulated DEGs (Fig. S8D). The ball-and-stick plot of the ‘Chondrocyte I’ DEGs revealed strong centrality and connectivity with a prominent supercluster including several themes: *Sarcomeres/Myofibrils Related*, *Amyloid-Beta Precursor Protein*, *BMP Signaling*, *Vertebral Compression Fractures*, and *Epiphyseal Dysplasia* (Fig. S9A, B). A small ribosome RNA-related supercluster comprising two themes *Cleavage in ITS2 between 5.8S RNA- and Mitochondrial lrRNA Export from Mitochondrion*, with a high enrichment score seen in *Mt-Atp8*, *Nxf1*, and *Nop53*. Other significantly altered genes, such as *App, Sost, Col1a1, Col1a2, Fgfr3*, and *Ttn*, indicated disruptions in cartilage biology, particularly in chondrocyte development and skeletal dysplasia. Collectively, these findings highlight a distinctive chondrocyte-like phenotype that is marked by alterations in growth factor signaling, endocytosis, and muscle-related pathways. In Chondrocyte I cells of *Mct1*^*cKO*^ mice, GSEA analysis showed increases in ‘positive regulation of gene expression’, ‘muscle contraction’, and ‘skeletal myofibril assembly’ (Fig. S8E, F).

**Fig. 6:**
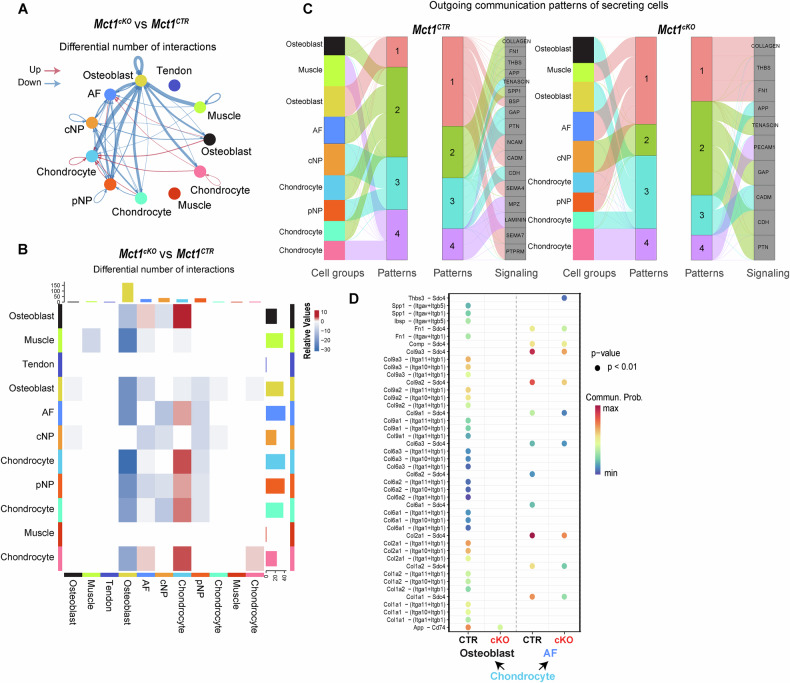
MCT1 deletion reduces intercellular communication in intervertebral discs. CellChat analysis of spatial transcriptomics data comparing communication activity between 6-month-old Mct1^CTR^ and Mct1^cKO^ mice. **A** Chord diagrams of differential ligand-receptor (L-R) interactions and the direction of altered interactions between cell types. Up-regulated and down-regulated interactions are indicated. **B** Heatmap of the number of interactions or total interaction strength (weights) between cell types in each genotype. **C** River plots of outgoing signaling patterns of secreting cells by cell type in each genotype. **D** Bubble plots of significantly altered L-R signaling pairs, with point size denoting significance level and color indicating communication probability.

Additionally, we conducted CellChat analysis to determine how MCT1 deletion affects intercellular communication within the spatial microenvironment of the disc [32]. Analysis of spatial transcriptomics data revealed that loss of MCT1 in collagen II-expressing cells reduced overall communication activity and rewired the organization of signaling networks within the spinal motion segment. Differential interaction mapping showed a substantial decrease in ligand–receptor in *Mct1*^cKO^ mice, particularly in interactions initiated by osteoblasts toward AF, chondrocyte populations, and other osteoblasts, while a subset of chondrocyte clusters exhibited modest increases in outgoing signaling (Fig. 6A). Corresponding heatmaps confirmed a global decline in both the number and relative strength of interactions across cell types in *Mct1*^*cKO*^ mice (Fig. 6B). River plots indicated a shift in the composition of functional signaling networks, with core ECM and adhesion pathways (COLLAGEN, THBS, FN1) largely preserved, whereas osteogenic (SPP1, BSP) and developmental guidance pathways (SEMA4, SEMA7, PATPRM, LAMININ) were lost. Notably, PECAM1 signaling uniquely emerged from chondrocyte III in cKO (Fig. 6C). Bubble plots further highlighted disrupted signaling from chondrocyte I to osteoblasts and AF populations (Fig. 6D). Together, these results indicate that MCT1 deletion leads to collapse of specialized osteogenic and developmental signaling, partial preservation of core ECM/adhesion cues, and emergence of reactive endothelial interactions – consistent with the impaired chondrocyte-to-osteoblast transdifferentiation and altered cellular identity observed within the immature bony endplate and adjacent NP in *Mct1*^cKO^ mice.

### EP chondrocytes but not AF cells utilize lactate as a metabolic fuel in the TCA cycle

**Fig. 7:**
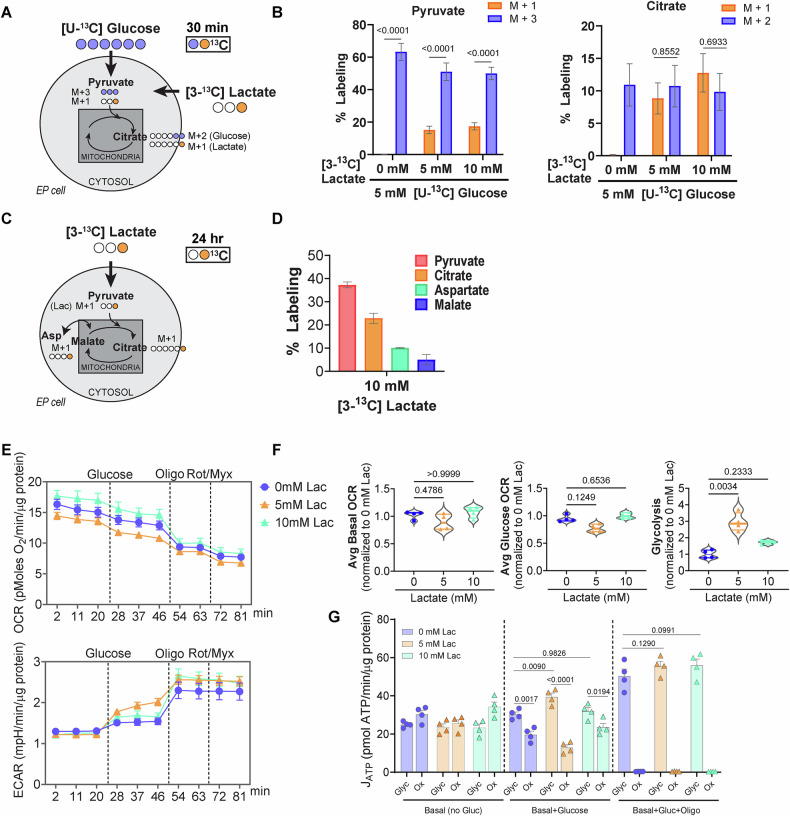
EP cells use lactate in the TCA cycle. **A** A schematic diagram of the dual [U-13C] glucose and [3-13 C] lactate tracing, demonstrating the flow of carbons into the TCA cycle. **B** Percent labeling of citrate and pyruvate after 30 min from 5 mM [U-13C] glucose with increasing concentrations of [3-13 C] lactate in EP cells. **C** Schematic diagram showing [3-13 C] lactate tracing, illustrating carbon flow into the TCA cycle. **D** Percent M + 1 labeling into TCA metabolites after 24 h from [3-13 C] lactate in presence of unlabeled glucose in EP cells, *n* = 5 independent samples/condition. Primary EP cells were cultured with sodium L-lactate for one week under osteogenic conditions to measure effect on bioenergetics. **E** Normalized ECAR and OCR traces, with corresponding **F** Average basal and glucose-dependent OCR, as well as glycolysis. **G** ATP production rate partitioned into glycolytic and oxidative ATP under basal, substrate, and ATP-linked conditions. Quantitative measurements are expressed as mean ± SEM (*n* = 4 experiments, with 4 technical repeats/group/trace) **E, G** and as violin plots with medians and interquartile ranges **F**. Significance was assessed using Kruskal-Wallis test with Dunn’s post hoc analysis **F** and one-way ANOVA with Sidak’s post hoc test **B, G**.

Lactate is converted to pyruvate by LDHB, which enters the TCA cycle and increases oxidative phosphorylation (OXPHOS). Although both glucose and exogenous lactate can generate pyruvate, the relative contributions of these two sources to EP chondrocyte metabolism are unknown. To address this, we isolated primary EP cells from rat intervertebral discs, which possess a distinct phenotypic profile (Fig. S10A). To determine the fate of lactate, we cultured these cells with [U-^13^C] glucose (5 mM) and increasing concentrations of [3-^13^C] lactate for 30 min to measure flux into M + 1 and M + 3 pyruvate and M + 1 and M + 2 citrate pools (Fig. 7A). We observed that approximately 50–60% of the pyruvate pool was derived from glucose (M + 3), while lactate contributed about 15-20% of pyruvate (M + 1) (Fig. 7B). Interestingly, glucose (M + 2) and lactate (M + 1) contributed about equally (~8-12%) to the citrate pool (Fig. 7B). These results suggest that in EP cells, a smaller fraction of glucose-derived pyruvate contributes to the citrate pool via the TCA cycle, whereas a significantly higher proportion of lactate-derived pyruvate enters into the TCA cycle. To assess lactate contribution to the metabolite pools at steady state, we performed a similar experiment where EP cells were cultured with [3-^13^C] lactate and unlabeled glucose for 24 h (Fig. 7C). We observed that there was further enrichment of labeling into pyruvate and citrate pools, and lactate also contributed to aspartate and malate pools (Fig. 7D). These experiments suggest that EP cells utilize both glucose and lactate as substrates, highlighting their mixed glycolytic and oxidative metabolism.

We performed a similar dual ^13^C-glucose and ^13^C-lactate labeling experiment to examine if AF cells utilize lactate (Fig. S11A). We observed that glucose was the major contributor to pyruvate (M + 3, ~66%), citrate (M + 2, ~15–20%), and lactate (M + 3, ~55%) pools. Only ~21% of the intracellular lactate was derived from tracer uptake (M + 1), and it contributed minimally to both pyruvate (M + 1, ~2–8%) and citrate (M + 1, ~1-5%) pools, suggesting that AF cells prefer glucose as their primary carbon source (Fig. S11B).

### Lactate does not alter metabolic substrate utilization in EP cells

To better understand the metabolic changes, we performed Seahorse assays to measure the rates of glycolytic and oxidative metabolism in EP chondrocytes cultured with varying amounts of lactate and under conditions that promote differentiation for one week [33]. Results showed that lactate supplementation was insufficient to drive major bioenergetic changes, except for increased glycolysis (Fig. 7E, F) and increased glycolytic ATP production in the 5 mM lactate group (Fig. 7G). Interestingly, this difference was lost with oligomycin treatment, suggesting the involvement of lactate-dependent mitochondrial activity.

To assess whether lactate promotes the utilization of glutamine or fatty acids in EP cells, substrate utilization was evaluated. There were no differences in OCR or ECAR traces, BPTES-dependent OCR, and glycolysis (Fig. S12A–C). Similarly, lactate did not appear to interfere with fatty acid utilization as confirmed by etomoxir (ETO) treatment (Fig. S12D, E). Taken together, these experiments suggest that lactate on its own or in the presence of glutamine or fatty acids does not alter common substrate utilization.

### MCT inhibition does not alter AF cell metabolism

To determine whether blocking lactate import via MCT1 affects AF cell metabolism, we next inhibited MCT1 using AZD3965 for 24 h under hypoxia. Seahorse assay with mixed-glucose-lactate substrates showed no significant changes in ATP production profiles or in the proportion of glycolytic versus oxidative ATP between control and MCT1-inhibited AF cells (Fig. S13A, B). Similarly, glycolytic capacity and reserve remained unchanged, indicating that lactate uptake or its absence does not alter AF cell substrate affinity and bioenergetics (Fig. S13C, D).

### Lactate promotes protein and histone lactylation in EP chondrocytes, altering their transcriptomic program

**Fig. 8:**
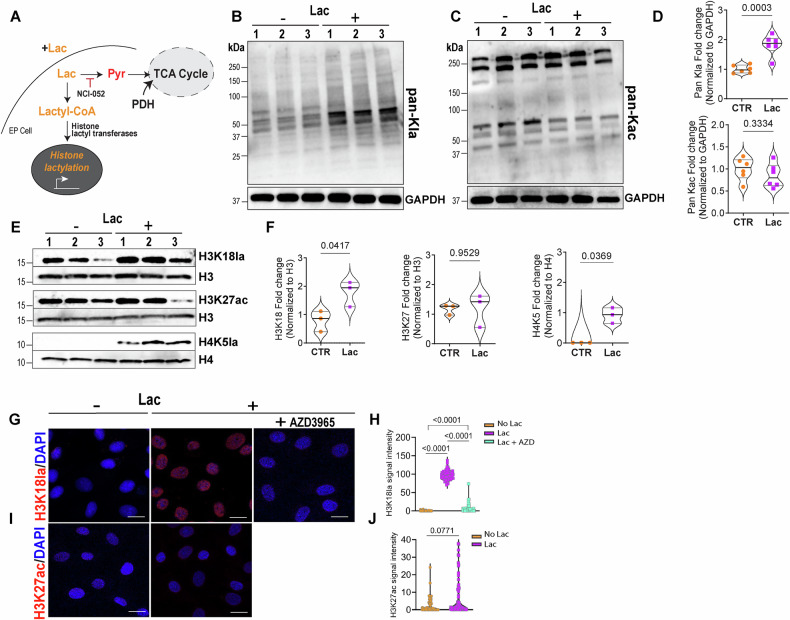
Lactate treatment in endplate cells induces lactylation. **A** A schematic diagram of lactylation via lactate-derived lactyl-CoA within an endplate cell. Lactate contributes to the formation of lactyl-CoA, which can be transferred to histones, by histones lactyl transferases, leading to histone lactylation, affecting gene expression. **B, C** Western blot of pan-lactylation (pan-Kla) and pan-acetylation (pan-Kac) in total protein from endplate cells treated with sodium L-lactate or NaCl for 24 h in physioxia. **D** Densitometric analysis of protein fold changes normalized to GAPDH, *n* = 6 independent samples/condition. **E, F** Western blot and densitometric analysis of histones extracted from lactate-treated endplate cells, probed for H3K18la, H3K27ac, and H4K5la, *n* = 3 independent samples/condition. Quantitative measurements are shown as violin plots with medians and interquartile ranges. **G, I** IF staining of H3K18la and H3K27ac in endplate cells treated with sodium L-lactate, NaCl, or MCT1 inhibitor, AZD3965 (500 nM), for 24 h in physioxia, scale bar = 15 μm. **H, J** Quantification of IF images as signal intensity (*n* = 70–80 cells/condition). Quantitative measurements are presented as violin plots, with medians and interquartile ranges shown. Original, uncropped western blots for all panels are available in the Supplementary Material.

To get the mechanistic insights into the diminished pan-Kla, H3K18-la and H4K5-la staining observed in *Mct1*^cKO^ mice, we sought to test whether lactate promoted protein and histone lactylation in EP cells (Fig. 8A). Lactate supplementation induced a clear increase in pan-lactylation with a negligible change in pan-acetylation (Fig. 8B–D). A similar pattern was observed with histone modifications, where there was a notable increase in lactylation of H3K18 and H4K5, with no differences in H3K27 acetylation (Fig. 8E, F). Similarly, immunofluorescence staining showed a prominent increase in nuclear staining of H3K18la following lactate treatment. Notably, control cells showed comparable H3K18la staining to cells that were pre-treated with MCT1 inhibitor, AZD, suggesting that MCT-1 dependent lactate import was necessary for the increased histone lactylation. Again, H3K27ac staining showed no discernible differences between lactate and control groups (Fig. 8G–J).

To explore the effects of lactate supplementation on the transcriptomic landscape in EP chondrocytes, we performed bulk RNA-Seq on cells treated with lactate for 24 h. There were 131 DEGs (FDR < 0.05, 80 down, 51 up), and hierarchical clustering showed a distinct clustering of samples in lactate-treated and untreated groups (Fig. S14A, B). We then investigated the level of expression of genes encoding key enzymes involved in lactylation, including a recently described lactyl-CoA synthetase, ACSS2, and several known lactyl transferases. The heatmap of TPM values shows a robust expression of *Acss2* by EP cells and a reduction following lactate-treatment. EP cells expressed several lysine acetyl-transferases (KAT) that serve as lactyl-transferases, including *Kat2a*, *Kat7*, and *Kat5*, with *Kat5* showing the highest expression (Fig. S14C). We analyzed the biological context of the upregulated and downregulated DEGs using the CompBio tool. Interestingly, analysis of the downregulated DEGs showed strong enrichment around *Very-long-chain Fatty Acyl-CoA oxidase activity*, *Fatty Acid Beta-Oxidation*, *Peroxisome Proliferator-Activated Receptor (PPAR) agonists*, *Insulin-like Growth Factor (IGF) Activity Regulation by IGFBPs*, *LPA/Lysophospholipid Signaling*, and *All-trans Retinoic Acid 18-hydroxylase Activity* (Fig. S15A). Strong gene signals from bone matrix protein, *Spp1*, were shown to be downregulated along with *Ankh*, suggesting decreased extracellular pyrophosphate. Other top genes within these thematic clusters include *Enpp2, Fads2*, *Acta1*, *Abca1*, *Scd*, *Igfbp5*, *Srebf1*, *Dhrs3 (All-trans Retinoic Acid 18-hydroxylase Activity)*, and *Mgll (Digestion of Cholesterol Esters)* (Fig. S15B). Key findings highlighted the roles of fatty acids, retinoic acid, pyrophosphate, calcium-dependent enzyme activities, and tissue development. Overall, the gene expression profile indicated lactate-mediated downregulation of fatty acid-related genes and suggested that the cartilage and sclerotomal phenotype was suppressed in endplate cells.

While upregulated DEGs showed slightly less pronounced thematic enrichment, many converged on pathways related to *Growth Factor Activity, Establishment of Mitotic Spindle Orientation Involved in Growth Plate Cartilage Chondrocyte Division, Axial Skeleton Plus Cranial Skeleton, Follistatin/Inhibin/Activin Axis, Mesenchymal Stem Cell Maintenance Involved in Nephron Morphogenesis* (Fig. S16A). Central ideas were focused on immune signaling pathways, inflammatory responses, and growth factor regulation, emphasizing molecular mechanisms that drive cell migration, activation, and replication inhibition. Significant changes in *Fgfr1*, *Csf1*, *Ccl2, Col2a1*, and *Meox1* suggested significant alterations in growth regulation and development (Fig. S16B). These findings revealed changes in the EP cell phenotype induced by lactate treatment.

In summary, our study, for the first time, highlights an MCT-dependent metabolic coupling between the EP, NP, and AF, wherein glucose and lactate serve as the primary metabolic currency. Consequently, in *Mct1*^cKO^ mice, the dysregulation of lactate coupling leads to impaired bony endplate formation, pronounced NP cell loss, and disc degeneration (Fig. S17A).

## Discussion

We have uncovered novel insights into the metabolic circuits regulating intervertebral disc function using a mouse model of conditional MCT1 deletion in *Col2a1*-expressing EP and AF cells (*Mct1*^*cKO*^). These studies were based on the growing recognition that lactate is more than just a metabolic waste product [25]. Instead, lactate has been shown to be a versatile metabolic fuel across various tissue types [20, 21]. Additionally, lactate functions as a signaling molecule that regulates gene expression through histone lactylation [22]. Our studies show that metabolic crosstalk mediated by lactate coupling between the disc compartments may contribute to the growth and overall health of the spinal motion segment.

Our in vivo studies highlighted the phenotype of *Mct1*^*cKO*,^ showing a striking delay in maturation of endplates, and persistence of hypertrophic cartilage in the region occupied by the bony endplate. By skeletal maturity, the endplate region develops into two distinct regions: the bony endplate, which overlays a thin, two-cell-thick layer of cartilage that persists throughout life. The bony endplate forms as mature chondrocytes transdifferentiate and are replaced by bone [5, 6]. Conversely, in *Mct1*^*cKO*^ mice, the persistence of endplate cartilage was evident by the absence of vascularization, the lack of osteogenic markers such as TNAP, and reduced mineralization. Furthermore, the robust COL X staining showed that the persisting chondrocytes were hypertrophic. Notably, *Mct1*^*cKO*^ mice phenocopied MCT4-KO mice, in which impaired lactate export from NP cells has been proposed to reduce lactate availability to adjacent endplate chondrocytes [9]. These observations are consistent with a functional role for lactate-mediated metabolic coupling between NP and EP cells. Accordingly, these disruptions in lactate-coupling in these MCT-mutants – acting directly rather than via lactate receptors, whose expression is minimal in EP cells – resulted in metabolic dysregulation and retardation of endplate differentiation, with chondrocytes unable to progress through the transdifferentiation process into the bone. Importantly, considering the comparable NP cellularity and proteoglycan matrix signatures between genotypes observed at earlier ages, which led to subsequent NP cell loss and degeneration in *Mct1*^cKO^ mice, suggested that the phenotype was unlikely to result from acute glucose deprivation alone. Instead, these findings are more consistent with chronic lactic acidosis, and structural alterations in the endplate may influence nutrient exchange. Persistent endplate avascularity could plausibly limit metabolite diffusion and clearance, although direct measurements of nutrient exchange were not performed in this study.

We employed spatial transcriptomics to gain deeper insights into the *Mct1*^*cKO*^ phenotype. Notably, the osteoblast cluster in the bony endplate was repositioned to an enthesis-like region in outer AF and at the NP/AF junction. This observation suggests that these cells may acquire an osteoblast-like fate, similar to that observed in *Ank*/*Ank* mice [34]. The ‘cNP’ and ‘Chondrocyte I’ clusters also showed pronounced changes in their signatures. Interestingly, recent scRNASeq studies have shown that NP cells in mice segregate into two distinct populations, ‘cNP’ and ‘pNP’, a finding consistent with our spatial results [35, 36]. These clusters showed greater divergence than other cell populations within the disc, underscoring their notochordal origin and unique transcriptomic profiles. Analysis of the ‘cNP’ cluster revealed disruptions in circadian clock concepts, which are essential for NP cell adaptation to their specialized niche [37]. Changes associated with NP degeneration were also reflected in altered expression of matrix-related genes such as *Acan* and *Sdc4* [38]. Additionally, alterations in endocytosis and Golgi apparatus function, involving genes such as *Cavin1* and *Golgb1*, have been implicated in disc degeneration [39, 40]. Intriguingly, GSEA analysis suggested enrichment of gene sets associated with muscle-like processes such as ‘actin-myosin filament sliding,’ ‘muscle filament sliding,’ and ‘muscle contraction.’ Importantly, these signatures are consistent with cytoskeletal remodeling or fibrotic responses rather than a true lineage transition, as several DEGs associated with these pathways are also linked to integrin signaling, focal adhesion dynamics, and regulation of actin-cytoskeletal organization. This provides a mechanistic link between MCT1 loss, disrupted lactate handling, and structural or cytoskeletal changes in NP cells, reflecting stress or fibrotic adaptation rather than a change in cell identity. Moreover, the ‘Chondrocyte I' cluster showed strong connectivity between themes relating to *Sarcomeres/Myofibrils Related*, and *BMP Signaling* with genes such as *App, Sost, Col1a1, Col1a2, Fgfr3*, and *Ttn*, indicating changes in muscle-related pathways and consequently, skeletal processes. GSEA further corroborated these outputs, revealing an upregulation in gene expression and metabolic pathways, indicating disruptions in cartilage matrix production and signaling. Recent studies have shown that PPARγ in osteoblasts regulates bone formation and fat mass by regulating sclerostin (SOST) expression; thus, we hypothesize that alterations in sclerostin activity could reflect changes in hypertrophic differentiation [41]. Similarly, FGFR3 plays a crucial role in inhibiting chondrocyte proliferation and differentiation, thereby regulating bone development. Mutations in *Fgfr3*, as seen in achondroplasia, lead to receptor over-activation, disrupting normal skeletal development and causing disproportionate short stature [42]. Furthermore, a study on the *Fgfr3*^*Y367C*^ activating mutation in skeletal stem/progenitor cells showed impaired bone healing, with these cells failing to support cartilage-to-bone transformation, leading to a pseudoarthrosis phenotype [43]. Consistent with these transcriptomic changes, CellChat analysis revealed shifts in intercellular communication and a reorganization of signaling networks in *Mct1*^cKO^ mice. These findings support the idea that the ‘Chondrocyte I’ cluster undergoes phenotypic alterations or disrupts cell-cell communication in chondrocyte differentiation. Moreover, the *App* gene, which is highly expressed in *Mct1*^*cKO*,^ was recently identified as a clusterin protein and may serve as a biomarker for osteoarthritis [44]. The emergence of PECAM1-associated signaling from chondrocyte III populations in *Mct1*^*cKO*^ mice, concurrent with reduced EMCN expression histologically, suggests that impaired lactate-mediated metabolic coupling may dysregulate vascular recruitment signaling during endplate ossification, though the precise mechanistic basis of this relationship warrants future investigation.

Previous in vitro work by Wang et al. showed that AF cells import lactate with about 10-15% of TCA cycle intermediates being derived from lactate [10]. Their conclusion that AF cells metabolize most of the NP-derived lactate, however, is paradoxical: the AF cells, like the NP cells, reside in an avascular, hypoxic environment and would be expected to generate lactate; thus, their reliance on this anion as an oxidative substrate is unexpected. To examine this possibility, we performed a [U-^13^C]-glucose and [3-^13^C]-lactate co-labeling experiment and found that glucose predominantly contributed to the pyruvate and citrate pools, indicating that glucose served as the primary substrate, with lactate playing a minor role. On the contrary, ^13^C stable isotope labeling experiments in EP cells revealed that lactate metabolism significantly contributed to TCA-derived metabolites, suggesting that EP cells rely both on glucose and lactate to replenish the pyruvate pool and utilize both substrates flexibly for TCA intermediate anaplerosis rather than ATP generation. This notion was further supported when bioenergetic profiles of EP cells showed a predominant reliance on glycolysis. Moreover, this glycolytic nature remained consistent even after 1-week exposure to lactate in the presence of other metabolic substrates like glutamine and fatty acids. Recently, studies by Thompson’s group demonstrated that lactate can suppress cancer cell glycolysis while inducing a metabolic shift towards OXPHOS [20]. However, given the absence of a strong OXPHOS shift, the mechanism appeared to be distinct from cancer cells or growth plate chondrocytes.

Based on lactate-dependent increases in pan-Kla, H3K18la, and H4K5la without change in pan-Kac, it was evident that exogenous lactate could induce lactylation in EP chondrocytes independently of acetylation. Recent studies have linked lactylation with histone acetyltransferases (HATs) or lysine acetyltransferases (KATs), such as HBO1 [24] and explored their effect on gene expression [22]. Our RNA-Seq analysis revealed a prominent downregulation in fatty acid genes, including *ACSS2*, a recently identified lactyl-CoA synthetase [45], and *KAT5*, the top-expressed KAT, along with *KAT2A*, which has been linked to lactyltransferase activity [46]. Further analysis revealed themes related to PPARγ, which is required for both adipocyte and cartilage differentiation; in particular, its downregulation here may play a critical role in chondrocyte differentiation [47]. A recent study of fracture healing showed the importance of PPAR signaling in regulating endochondral bone and vascular development, linking skeletal regeneration to metabolic and transcriptional pathways [48]. Similarly, downregulation of *Ankh*, *Enpp1*, and *LPA/Lysophospholipid Signaling* suggested altered pyrophosphate activity, and Enpp1 is regulated by H3K18la in bone marrow stromal cells [49, 50]. Pyrophosphate is a crucial regulator of bone mineralization as it inhibits hydroxyapatite crystal formation and growth. Moreover, we have shown that *Ank* loss leads to abnormal mineralization and the transformation of AF cells into an osteoblast-like phenotype [34, 51]. In terms of lipid metabolism, we observed a predominance of fatty acid-related themes, such as *Fads*, indicating a downregulation in phospholipid or fatty acid metabolism. This observation was further supported by a suppression in cholesterol-related genes *Mgll, Srebf1, Scd*, which are key players in phospholipid metabolism. It should be acknowledged that phospholipids are essential in regulating chondrocyte maturation, notably through the Wnt/β catenin pathway, and in maintaining the integrity of the ECM [52]. Beyond structural functions, phospholipids actively participate in signaling processes that drive chondrocyte differentiation and maturation. Of the hormones that regulate cartilage homeostasis and maturation, one of the most important is IGF-1 [53]. The observed changes in *Igfbp5*, a classical IGF-binding protein, suggest a shift in IGF signaling and, subsequently, altered chondrocyte maturation [54].

Computational analysis of the upregulated DEGs indicated changes in growth regulation and development. The upregulation of *Fgfr1* and *Col2a1* suggested a transition from a proliferative to a differentiated state. A previous study demonstrated that FGFR1 signaling in hypertrophic chondrocytes plays a critical role in endochondral bone formation through a complex intracellular mechanism involving neurofibromin [55]. Upregulation of *Csf1*, *Ccl2*, and *Meox1* may further support chondrocyte maturation by modulating inflammatory and immune responses that typically drive cell proliferation and migration, thereby creating a more stable, controlled environment conducive to proper chondrocyte differentiation. In this context, upregulation of excessive growth factor signaling and inflammatory pathways likely aids in the orderly progression of chondrocytes through their maturation stages, contributing to more organized cartilage formation. Among upregulated DEGs, *Serpine1*, in addition to *Col2a1*, has been linked to lactate-responsive histone lactylation in other systems, while *Inhba* has established roles in chondrocyte growth regulation and differentiation, respectively [56, 57]. It is, however, important to note that genome-wide CUT&Tag studies show that H3K18la marks promoters and enhancers in a highly tissue-specific manner, and this information is currently unavailable for EP cells [58]. Together, these findings suggest potential mechanistic connections between lactate signaling, epigenetic regulation, and chondrocyte maturation, although their functional roles in EP cells remain to be established. Overall, lactate treatment induces a complex array of changes that appear to promote a hypertrophic phenotype, while suppressing the cartilage sclerotomal phenotype.

In summary, our study reveals a novel potential role for lactate in growth-dependent metabolic coupling between the endplates and other disc compartments. Beyond its role as a metabolic fuel, lactate may function as a signaling molecule in disc cells, influencing gene expression and driving chondrocyte maturation through lactylation. While direct demonstration of lactylation’s functional role in disc cells would provide valuable mechanistic insight, the precise molecular machinery mediating protein and histone lactylation in disc cells remains incompletely undefined and consequently extends beyond the scope of the present work and represents an important direction for future investigations. While direct demonstration of lactate flux between disc compartments in vivo will be useful for fully establishing the proposed metabolic coupling model, such measurements remain technically challenging due to the limited vascularity of the intervertebral disc and the small size of the endplate compartment, which limits the ability to perform compartment-specific metabolic tracing in vivo. In summary, our findings highlight the importance of lactate in disc biology and suggest its potential as a biomarker of normal disc function, warranting further investigation into its role in disc health and disease.

## Materials and methods

### Mice

All animal studies were approved by the Institutional Animal Care and Use Committee (IACUC) of Thomas Jefferson University. *Mct1* conditional knockout (*Mct1*^*cKO*^: *Col2a1*^*CreERT2*^*Mct1*^*f/f*^) and control (*Mct1*^*CTR*^: *Mct1*^*f/f*^) mice were generated by crossing *Mct1*^f/f^ mice with *Col2a1*^CreERT2^ mice [26, 27] (Fig. 1A). For all experiments, 2-week-old female and male mice of all genotypes received an intraperitoneal injection of 100μg/g body weight of tamoxifen (Sigma-Aldrich, St. Louis, MO, USA) dissolved in corn oil (Sigma-Aldrich) for 5 consecutive days to activate Cre recombinase and were analyzed at 10-weeks, 16-weeks, 6-months, and 12-months to investigate the effects of *Mct1* loss on disc health. To visualize Cre targeting, a loxP-stop-loxP tdTomato reporter mouse Ai9 (Strain #007909, Jackson Labs) was crossed with mice with (*Col2a1*^*CreERT2*^*Mct1*^*f/f*^) or without (*Col2a1*^*CreERT2*^) conditional *Mct1* loss.

### Histological analysis

Dissected spines were immediately fixed in 4% paraformaldehyde for 4 or 24 h to process for frozen or paraffin embedding, respectively, following decalcification in 20% EDTA at 4^o^C. 7-μm-thick mid-coronal sections were stained with 1% Safranin-O, 0.05% Fast Green, and 1% Hematoxylin to assess morphology and imaged on an Axio Imager A2 microscope using 5x/0.15 N-Achroplan or 20x/0.5 EC Plan-Neofluar (Carl Zeiss) objectives, Axiocam 105 color camera, and Zen2™ software (Carl Zeiss AG, Germany) [31]. Disc degeneration was evaluated using three blinded observers and histological scoring was performed using a Modified Thompson grading scale for the NP and AF, and the Tessier endplate grading scale for the EP [59] histological scores for each disc. Since the unique interactions between genetic, biological, and biomechanical factors at individual spinal levels have been shown to produce different phenotypic outcomes, each disc was considered as an independent sample [60–62]. Histological grades (grades 1–5) increase with the severity of degeneration.

The quantification of the percentage of Safranin-O-positive stained area in the 12-month endplates (EP) was conducted using the Fiji package of ImageJ. The colors of the images were separated using the “Split channels” function, and manual thresholding was done on the red channel of the images. Compartments of the superior EP and inferior EP were then manually selected using the “Polygon Selection” tool, and the positively stained area was analyzed using the Measure function and Area Fraction measurement.

Hindlimbs from 6-month-old *Mct1*^*CTR*^ and *Mct1*^*cKO*^ were decalcified in 19% EDTA for 21 days, followed by processing, paraffin embedding, and coronal sectioning, as described previously [63]. Mid-coronal sections were stained with H&E on the medial tibial plateaus (MTP) and femoral condyles (MFC) to assess cartilage osteoarthritis (OA) damage [40].

### TUNEL staining

The TUNEL assay was performed on disc tissue sections from 12-month-old *Mct1* mice using an “In Situ Cell Death Detection” kit (Roche Diagnostics). Briefly, sections were deparaffinized and permeabilized with Proteinase K (20 μg/mL) for 15 min at room temperature. The assay was then conducted following the manufacturer’s instructions, after which the sections were mounted with ProLong® Gold Antifade Mountant containing DAPI (Thermo Fisher Scientific, P36934). Mounted slides were imaged using an Axio Imager 2 microscope, as previously described [31]. TUNEL-positive cells and DAPI-positive cells were quantified using ImageJ to assess cell death and cell number in the disc compartments, respectively.

### Picrosirius red analysis

Picrosirius Red staining on disc sections of 12-month-old mice was performed to visualize collagen fibril thickness. A polarized light microscope was used to visualize collagen organization, as previously described [31]. Under polarized light, collagen fibrils are visualized as green, yellow, or red pixels corresponding to thin, intermediate, or thick fibrils. The color threshold levels were kept constant throughout the analysis.

### Immunohistochemistry

Mid-coronal 7-μm paraffin from 12-month-old mice were deparaffinized in Histoclear and rehydrated in a graded ethanol series before antigen retrieval using one of the following methods: a 30-min incubation in hot citrate buffer, a 10-min incubation at room temperature with proteinase K, or a 30-min incubation at 37°C with Chondroitinase ABC. Following antigen retrieval, the sections were blocked with 10% normal goat serum (Thermo Fisher Scientific, 10,000 C) in PBS-T (0.4% Triton X-100 in PBS), and incubated with primary antibodies against MCT1 (1:150; Invitrogen; PA5-72957), GLUT1 (1:100; Abcam; ab115730), MCT4 (1:100; Invitrogen; PA5-87977), COL X (1:500; Abcam; ab58632), EMCN (1:100; Santa Cruz; sc65495), TNAP (1:50; sc271431), COL 1 (1:100; Millipore Sigma; ABT123), ACAN (1:50; Millipore Sigma; AB1031), C.S. (1:300; Abcam; ab11570). Similarly, some discs embedded in OCT were sectioned on CryoStar™ NX70 Cryostat and briefly fixed with acetone. Tissue sections were then blocked with 10% serum (Thermo Fisher Scientific, 10,000 C) in PBS-T or M.O.M.™ Immunodetection Kit (Vector Laboratories, BMK-2202), and then incubated with primary antibodies against LDHA (1:100; Novus; NBP2-19320), LDHB (1:50; Santa Cruz; sc100775), pan-Kla (1:100, PTM Bio, PTM-1401-RM), H3K18la (1:100, PTM Bio, PTM-1406RM), H4K5la (1:100, PTM Bio, PTM-1407RM), and total Histone H3 (1:100; CS; 4499). Sections were washed and incubated with Alexa Fluor-488 or -594 conjugated secondary antibodies (1:700, Jackson ImmunoResearch, West Grove, PA). for 1 h at room temperature. In addition, F-CHP (3Helix) assay was performed following the manufacturer’s protocol. Following incubation, sections were washed, mounted, and imaged on Axio Imager 2 microscope (Carl Zeiss) using 5x/0.15 N-Achroplan or 10x/0.3 EC Plan-Neofluar (Carl Zeiss) objectives, X-Cite 120Q Excitation Light Source (Excelitas) and AxioCam MRm R3 camera (Carl Zeiss Microscopy) or Zeiss LSM 800 Axio Inverted confocal microscope with Plan-Apochromat 20x/0.8 and Zen 2 (blue edition) software (Carl Zeiss Microscopy). Quantification was conducted in greyscale using ImageJ.

### Micro-computed tomography (μCT) Analysis

Spines of 12-month-old *Mct1*^*CTR*^ and *Mct1*^*cKO*^ mice were placed in PBS and scanned on μCT scanner (Skyscan 1272, Bruker, Belgium) at an energy of 50 kVp, current of 200 μA, and a 10 μm^3^ voxel size resolution. In DataViewer, length of vertebral bones and height of adjacent discs were measured in the dorsal, midline, and ventral regions of the sagittal plane and averaged; from this, disc height index (DHI) was calculated as previously described [31]. Cortical and trabecular bone microstructure was also analyzed from these scans, and the following parameters were analyzed: trabecular separation (Tb. Sp.), trabecular thickness (Tb. Th.), trabecular number (Tb. N.), trabecular bone volume fraction (BV/TV), and trabecular bone mineral density (Trab. BMD). Cortical bone mineral density (Cort. BMD), mean cross-sectional bone thickness (Cs. Th.), cortical BV, and mean cross sectional bone area for the cortical bone. The bone mineral density (BMD) of the caudal endplates was tabulated in a region of interest (ROI) defined by outlining the superior and inferior bony endplates that lie below the growth plate, as previously described [34].

Mouse hindlimbs were harvested and fixed for analysis. MicroCT imaging of the knee joint was performed using a Bruker SkyScan 1275 scanner, as previously described [63]. Quantitative assessment of the tibial subchondral bone — including bone volume fraction (BV/TV), trabecular thickness (Tb.Th), trabecular separation (Tb.Sp), and subchondral bone plate thickness (SCBP) —was conducted on coronal slices encompassing the medial and lateral tibial plateaus [40].

### Spatial transcriptomics

FFPE blocks of *Mct1*^*CTR*^ and *Mct1*^*cKO*^ mice were generated, embedding one caudal (Ca7/8) level from four different mice into each paraffin block. Spatial transcriptomic profiling was performed at 6 months as a time point immediately after skeletal maturity and the earliest stage at which endplate and NP tissue structural phenotypes are fully detectable while preserving tissue integrity for VisiumHD analysis [64]. Following RNA quality assessment (DV200 > 30), 5-μm sections were cut and placed onto Nexterion Slide H – 3D Hydrogel Coated slides. Hematoxylin and Eosin (H&E) staining was performed to orient the tissue sections. The slides were loaded into the Visium CytAssist system to facilitate the transfer of oligonucleotide barcodes from the Visium slides to the tissue sections. The Visium CytAssist Gene Expression for FFPE workflow was employed to automate the transfer of transcriptomic probes from glass slides to Visium slides. Tissue permeabilization was optimized by increasing the decrosslinking temperature from 70°C to 95°C to enhance probe access to genomic DNA (gDNA). This adjustment improved the detection of Unique Molecular Identifier (UMI) counts from both gDNA and mRNA probe ligation events. Following barcode transfer, the barcodes were collected, and sequencing libraries were prepared using the NovaSeq-Sp100 kit, following the manufacturer’s protocol.

Raw reads from Visium libraries were processed using SpaceRanger v3.0.0. Associated CytAssist and H&E images were manually aligned using Loupe v8.0.0. Raw gene counts from 8 μm bins were further processed using Seurat v5.0.3. Bins with less than 20 counts or 20 observed genes were discarded. To form bins larger than 16 μm, an array with the coordinates and gene expression values from the 16 μm binned data was convolved using a matrix of ones and size equal to the desired binning multiple. Then, rows and columns were dropped such that none of the convolved 16 μm bins overlap. Associated spatial metadata was recalculated by linear transformations of the 16 μm metadata using the combining multiplier. For this analysis, 48 μm bins were generated and clusters were identified using the FindClusters function in Seurat with default parameters and Resolution=1.0. Cluster annotation was manually performed by analyzing the top marker DEGs in Seurat for each cluster.

Cluster-specific differential gene expression analysis was conducted across control and experimental conditions with spatial spots aggregated over all replicates. Genes were filtered for having at least 10% of spatial spots expressed in one condition. Statistically significant differentially expressed genes (DEGs) were found using the FindMarkers function in Seurat v5.0.3 with MAST, with *q* < 0.05 after FDR correction for multiple comparisons. Pathway analyses were performed for DEGs per cluster using the prerank function on average log2 fold change with default parameters in GSEApy v1.1.3. The GO Biological Processes 2021 gene set library was used as reference. Significant overlap of ranked DEGs with pathways was depicted both at *q*-value of 0.25 as recommended by GSEA documentation and at *q*-value of 0.05, after FDR correction. The normalized enrichment score was used to assess directionality of association between DEGs and pathways - a positive score denotes the genes at the top of the ranked list (upregulated genes) as having significant overlap with the pathway, whereas a negative score corresponds to the bottom of the ranked list (downregulated genes). Spatial sequencing data is deposited in the GEO database (GSE287499).

### CellChat analysis

Spatial transcriptomic data from intervertebral disc endplates of 6-month-old *Mct1*^*CTR*^ and *Mct1*^*cKO*^ mice were analyzed using CellChat to infer intercellular communication networks based on known ligand–receptor interactions. Spatially resolved gene expression profiles were used to identify overexpressed signaling molecules and estimate communication probabilities between neighboring cell populations. Aggregated networks were compared between genotypes to assess changes in interaction strength and pathway activity. Differentially regulated interactions were visualized as chord diagrams, heatmaps, river plots, and bubble plots in R [32].

### CompBio analysis

Spatial transcriptomics data from *Mct1*^*CTR*^ and *Mct1*^*cKO*^ mice, along with bulk RNA-Seq data were analyzed using the GTAC-CompBio Analysis Tool (PercayAI Inc., St. Louis, MO). CompBio employs the Biological Knowledge Generation Engine (BKGE) to extract PubMed abstracts referencing input differentially expressed genes (DEGs) and identify relevant biological processes and pathways. Conditional probability analysis was applied to compute enrichment scores, which were normalized against randomized query groups to determine statistical significance. Related concepts derived from DEGs are grouped into broader, higher-level themes, which represent overarching biological patterns, such as pathways, processes, cell types, and structures. The Normalized Enrichment Score (NEScore) quantifies enrichment magnitude, and results were clustered based on fold enrichment and rarity as previously described [31].

### Primary annulus fibrosus (AF) and endplate (EP) cell isolation

Primary AF and EP cells were harvested and cultured from adult Sprague Dawley rats. AF cells were supplemented with DMEM containing 10% FBS. To investigate the effects of lactate on cell metabolism [65], AF cells were cultured at 5% O_2_ in a hypoxia workstation (InvivO_2_ 300; Baker Ruskinn, USA) with or without sodium L-lactate for 24 h. Primary rat EP cells were harvested and cultured from 3-week-old Sprague Dawley rats [66]. Isolation protocols differed notably in enzyme concentrations and incubation times, with Pronase used at a 2:1 ratio and Collagenase P at a 29:1 ratio for EP:AF. Following enzymatic digestion, cell suspensions are passed through a 70 µm sterile strainer to enrich for EP cells. These cells were further cultured in 5% O_2_. For the cell differentiation studies, EP cells were cultured in complete DMEM with 10 mM β-glycerophosphate (Sigma-Aldrich, G9422), 50 μg/ml L-ascorbate-2-phosphate, and 1x insulin, transferrin, selenium (Gibco, 41400045) and varying concentrations sodium L-lactate up to 7 days under 5% O_2_ (InvivO_2_ 300; Baker Ruskinn, USA). To measure protein lactylation, EP cells were cultured in DMEM supplemented with 20 mM sodium lactate or 20 mM NaCl for 24 h.

### Glucose and lactate stable isotope tracing analysis

Primary AF (*n* = 4 sets/condition) and EP (*n* = 5 sets/condition) cells were plated in 6 cm dishes at a density of ~10^6^ cells and incubated in 5% O_2_ for 24 h prior to metabolomic analysis the following day. All experiments were completed in DMEM media without glucose, glutamine, phenol red, sodium pyruvate, and sodium bicarbonate (Sigma, D5030) that was supplemented with 44 mM sodium bicarbonate, 4 mM glutamine, 5 mM glucose, and 10% dialyzed FBS (Corning 35-071-CV). The next day, the cultures were replenished with media containing 5 mM [U-^13^C] glucose (Cambridge Isotope Laboratories, CLM-1396) and varying concentrations up to 10 mM [3-^13^C] lactate (Cambridge Isotope, CLM-1579) as isotope tracers and incubated for 30 mins to limit the lactate contribution to the second turn of the TCA cycle. Another experiment (*n* = 5 sets) was conducted in EP cells treated with 10 mM [3-^13^C] lactate and 5 mM unlabeled glucose for 24 h in hypoxia to determine where the lactate pool accumulates (shown by percent enrichment of labeled isotopologue M + 1 labeling) Cells were harvested and polar metabolites were extracted by the addition of 1 ml ice-cold extraction solution of 80% methanol/20% water. Unlabeled samples were also prepared for each experiment, from which an unlabeled sample pool was generated. Metabolomics analyses were performed by The Wistar Institute Proteomics and Metabolomics Shared Resource. Samples were analyzed by LC-MS/MS on Thermo Scientific Q Exactive HF-X or Plus mass spectrometers interfaced with Vanquish UHPLC Systems. Samples were analyzed in a pseudorandomized order.

LC separation was performed using a ZIC-pHILIC column, 150 × 2.1 mm, 5 μM (EMD Millipore) maintained at 45 °C. Mobile phase A was 20 mM ammonium carbonate, 5 µM medronic acid, 0.1% ammonium hydroxide, pH 9.2, and mobile phase B was acetonitrile. Analytical separation was performed at 0.2 ml/min flow rate using the following step-wise gradient: 0 min, 85% B; 2 min, 85% B; 17 min, 20% B; 17.1 min, 85% B; and 26 min, 85% B. Samples were analyzed by either Full MS scans with polarity switching (all samples) or Full MS scans with data-dependent MS/MS scans with separate acquisitions for positive and negative polarities (unlabeled sample pool). Relevant MS parameters include: sheath gas, 40; auxiliary gas, 10; sweep gas, 2; auxiliary gas heater temperature, 350°C; spray voltage, 3.5/3.2 kV for positive/negative polarities; capillary temperature, 325°C; S-lens RF, 40 for HF-X and 50 for Plus. Full MS scans were acquired using a scan range of 65 to 975 m/z; resolution of 120,000 for HF-X and 70,000 for Plus; automated gain control (AGC) target of 1E6; and maximum injection time (IT) of 100 ms. Data-dependent MS/MS was performed on top 10 ions; resolution of 15,000 for HF-X and 17,500 for Plus; AGC target of 5E4, maximum IT of 50 ms, isolation width of 1.0 m/z, and a stepped normalized collision energy of 20, 40, 60.

Raw data were analyzed using Compound Discoverer 3.3 SP3 (Thermo Scientific). Metabolites were identified in the unlabeled sample pool by accurate mass in conjunction with either retention time based on pure standards or MS/MS fragmentation by querying the mzCloud database. These identifications were applied to labeled samples with consideration for all possible ^13^C isotopologues. Metabolite levels were quantified by integrating peak areas from Full MS data, and data were corrected for natural ^13^C abundance and tracer purity [67].

### Seahorse metabolic assays

Primary rat EP cells were plated in Seahorse microplates at appropriate densities (15,000 cells/well for 24-h experiments and 4000 cells/well for long-term experiments) and were allowed to adhere overnight to 2 days. Cell culture media was then replenished with or without sodium L-lactate under 5% O_2_ for 24 h, or up to 7 days prior to Seahorse measurements. Prior to analysis, cells were washed twice with Krebs-Ringer-Phosphate-Hepes (KRPH) buffer and incubated in KRPH + 0.1% BSA with or without lactate. The microplates were degassed for 1 h before placing the cells in a Seahorse Flux Analyzer. For ATP consumption assays, cells are sequentially injected with 5 mM glucose, 2 μg/ml oligomycin, 1 μΜ rotenone with 1μΜ myxothiazol while monitoring changes in oxygen consumption rate (OCR) and extracellular acidification rate (ECAR). For substrate utilization assays, cells were sequentially injected with 5 mM glucose with 4 mM glutamine or palmitate-BSA (palmitate concentration 150 μM); 2 μg/ml oligomycin, 1 μΜ FCCP followed by 5 μΜ BPTES (GLS inhibitor) or 5 μΜ Etomoxir (CPT1 inhibitor). Raw ECAR and OCR raw traces were plotted and used to quantify ATP production – specifically, the partitioning of glycolytic and oxidative ATP [68, 69]. Parameters relating to glycolysis (glucose-stimulated minus basal), glycolytic capacity (after oligomycin addition), and reserve capacity (oligomycin-stimulated minus basal) were extracted from ECAR values. OCR values were used to determine ATP-coupled respiration (after oligomycin addition), endogenous (no substrate), and basal (after substrate addition) as previously described [1]. For additional assays, AF cells were treated with MCT1 inhibitor, AZD3965 (Sigma AMBH2D6FA3BB), either at 500 nM or 1 μM, for 24 h, and ATP consumption and glycolytic capacity were probed in a mixed glucose-lactate environment. For both assays, cells are subsequently injected with 5 mM glucose and 7.5 mM sodium lactate. For glycolytic capacity, 1 μΜ Rotenone & 1 μΜ Myxothiazol; 1 μΜ FCCP and 200 μΜ Monensin injections succeeded the substrate injection. ECAR and OCR raw traces were generated, and the proton production rate was used to quantify glycolytic capacity: basal, ATP-demand rate (+ Rotenone and Myxothiazol), maximal glycolytic capacity, and reserve [68, 69]. Results were normalized to total protein.

### Western blot

Treated and untreated EP cells were lysed, and 35 μg of whole cell lysate or 15 μg of purified histones (Abcam, ab113476), were electroblotted to PVDF membranes (EMD Millipore, IPVH00010). The membranes were blocked with 4% (w/v) nonfat dry milk in TBS-T and incubated overnight at 4°C with antibodies against pan-Kla (1:1000; PTM-1401) pan-Kac (1:1000; PTM-101), H3K18la (1:1000; PTM-1406RM), H3K27ac (1:1000; PTM-116RM), H4K5la (1:1000, PTM-1407) and housekeeping antibodies against GAPDH (1:1000; CS), total Histone H3 (1:3000; CS; 4499), or Histone H4 (1:1250; Abcam; ab7311). An ECL reagent (Prometheus, 20-301) was utilized to detect immunolabeling using an Azure 300 system. Densitometric analysis was conducted using ImageJ software.

### Immunocytochemistry

EP cells were plated on collagen I-coated glass coverslips and treated for 24 h under hypoxic conditions. Cells were then fixed with ice cold methanol for 10 min., permeabilized with 0.1% Triton X-100 for 15 min. and blocked with 1% BSA for 1 h. Cells were incubated overnight at 4°C with primary antibodies against H3K18la (1:1000; PTM-1406RM) and H3K27ac (1:1000; PTM-116RM), diluted in blocking buffer. After washing, cells were incubated with Alexa Fluor-594-conjugated secondary antibodies and mounted with ProLong Gold Antifade Mountant with DAPI. Negative controls were used to confirm the specificity of staining. Cells were visualized using a Zeiss LSM 800 Axio Inverted confocal microscope (Plan-Apochromat 40x/1.3 oil or 63x/1.40 oil). Quantification was conducted in greyscale using ImageJ (version 1.54 g) [33].

### RNA-seq

DNA-free RNA was isolated from treated endplate cells using a RNeasy^®^ Micro kit (Qiagen, Venlo, Netherlands). RNA quality and concentration were assessed using a Nanodrop ND 100 spectrophotometer (Thermo Fisher Scientific) and an Agilent 2200 Tape Station (Agilent Technologies, Palo Alto, CA, USA), respectively. cDNA was synthesized using the RNA to cDNA EcoDry Premix (Takara, #639549) and subsequently used for qPCR validation. For bulk RNA sequencing, purified RNA was sent to Azenta Life Sciences/Genewiz (Chelmsford, MA, USA). Library preparation, including poly(A) enrichment and cDNA synthesis, was performed by Azenta according to their standard protocols. Sequencing was conducted on an Illumina platform, generating paired-end reads with a length of 150 bp. Raw sequencing data underwent quality control and were provided as FASTQ files for bioinformatic analysis. Differential gene expression was performed using a threshold of an adjusted *p*-value (FDR) < 0.05 and log2 fold change cutoff to identify significant changes in expression. Spatial sequencing data is available in the GEO database (GSE287056).

### Statistical analysis

Statistical analysis was performed using Prism 10 (GraphPad, La Jolla, CA) with data presented as mean +/- standard error mean to represent the precision of the mean estimate. Normality was assessed using the Shapiro-Wilk tests to evaluate differences between distributions. For normally distributed data, an unpaired t-test was used for comparisons, while the Mann-Whitney U test was applied for non-normally distributed data. To compare multiple distributions of non-normally distributed data, the Kruskal-Wallis test with Dunn’s multiple comparison was used. For normally distributed data, one-way ANOVA with Sidak’s post-hoc multiple comparison test was used. The distributions of Modified Thompson Grading and fiber thickness data were analyzed using a Fisher’s exact test or χ² test with a significance level set at 0.05. All tests were two-tailed tests.

## Supplementary information

Supplementary Figures (S1–S17)

Original Western Blots

Supplementary Table

## Data Availability

Spatial transcriptomics and bulk RNA-Seq data associated with this study are deposited in the GEO database under the accession codes: GSE287499 and GSE287056, respectively. All generated and analyzed data are included in this publication.
