# Supplementary material for: Loss of MCT1 mediated lactate uptake causes delayed endplate maturation and intervertebral disc degeneration
Source: Cell Death Dis. 2026 Jun 3;17(1):698. doi: 10.1038/s41419-026-08942-4 (PMC13448696; doi:10.1038/s41419-026-08942-4)
Supplement: Supplementary file 1 [file 41419_2026_8942_MOESM1_ESM.pdf]

Supplementary Figure 1

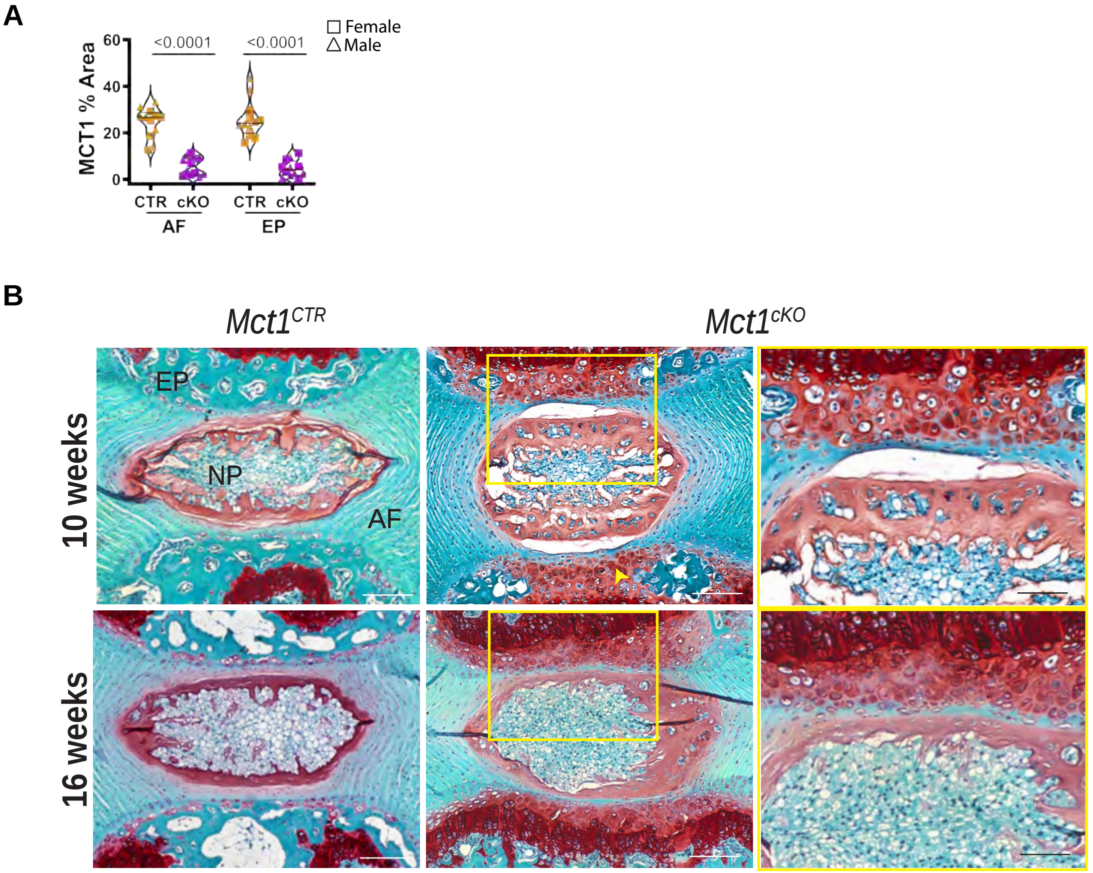

Supplementary Figure 1. **Early manifestations of Mct1^{cKO}-induced disc and endplate alterations.** A) Quantification of MCT1 immunohistological stain of Mct1^{cKO} and Mct1^{CTR} mice: n = 4 mice/genotype (2 males, 2 females); 3 discs/ mouse. Data represented as violin plots with median and quartiles. Significance was determined using an unpaired t-test based on normality. B) Safranin O/Fast Green staining of the discs from 10- and 16-week-old Mct1^{CTR} and Mct1^{cKO} mice. n = 2 mice/genotype; 3 discs/mouse. Yellow arrowheads indicate degenerative changes. Scale bar: Columns 1 and 2 = 100 μm; Column 3 = 50 μm.

Supplementary Figure 2

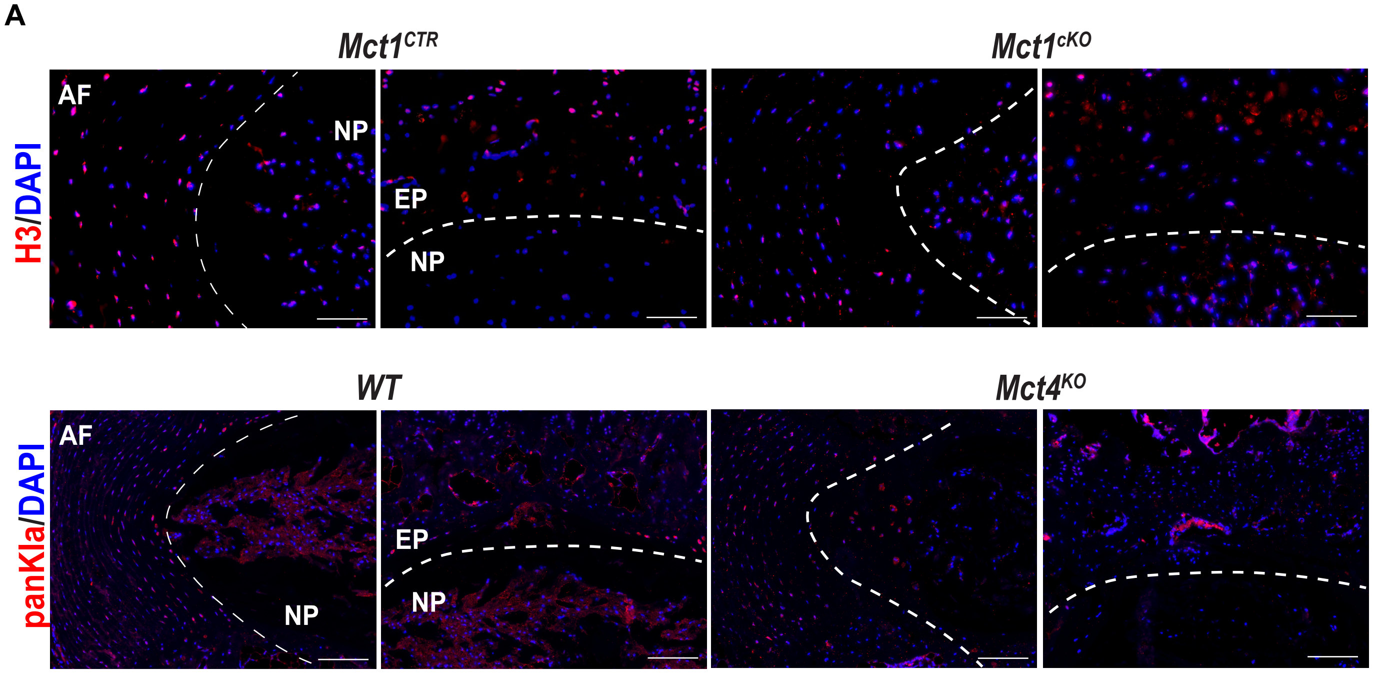

Supplementary Figure 2. **MCT loss affects lactylation in the disc**. A) Representative images showing total H3 in the EP, AF, and NP compartments of 6-month-old Mct1^{CTR} and Mct1^{cKO} mice, scale bar = 50 µm. n = 2 mice/genotype (1 male, 1 female), 4 discs/ animal assessed. B) Representative images of pan-Kla in the AF-NP and EP of 14-month-old WT and Mct4^{KO} mice, scale bar = 100 µm, n= 3 mice/genotype (2m, 1f), 2-3 discs/mouse. Dotted lines indicate boundaries between different tissue compartments within the disc.

Supplementary Figure 3

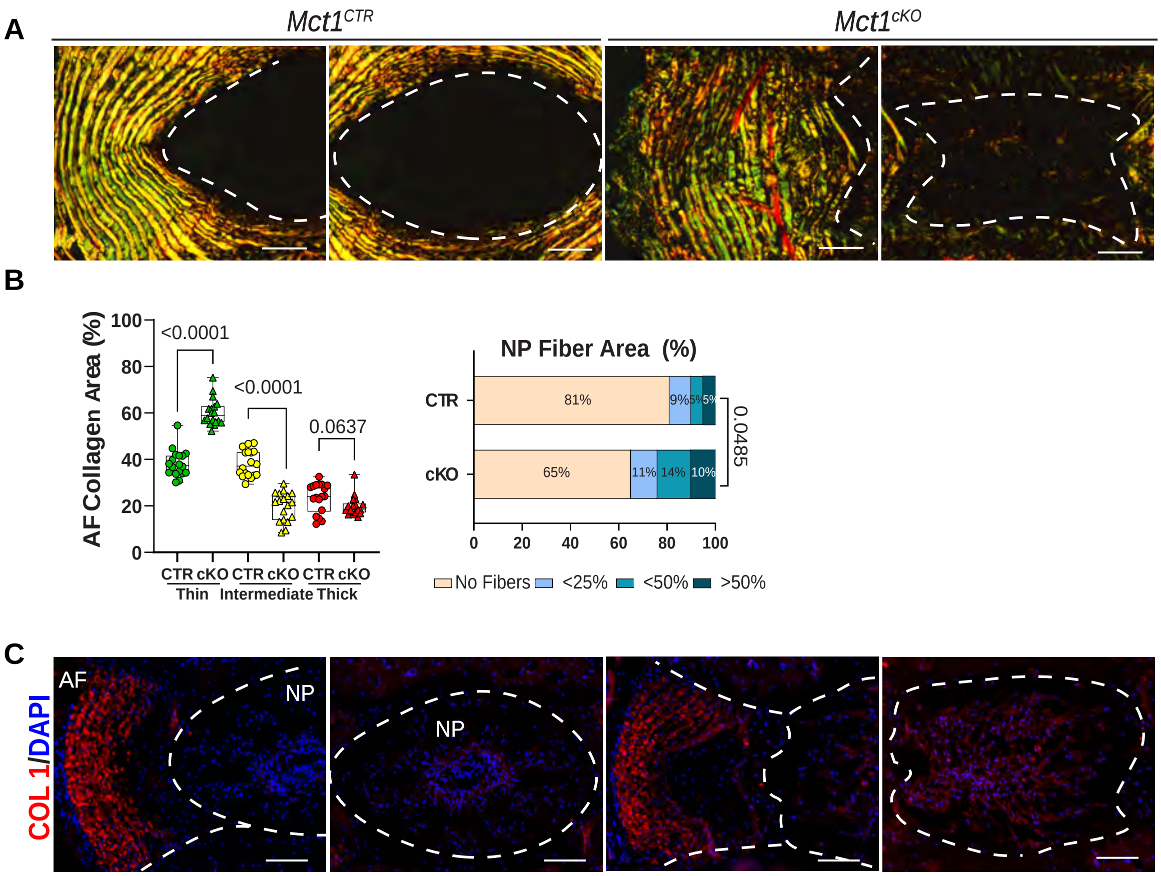

Supplementary Figure 3. **Mct1cKO discs exhibit disorganized matrix and NP fibrosis.** A) Polarized imaging of picrosirius red stained discs of 12-month-old Mct1CTR and Mct1cKO mice showing collagen organization, scale bar = 100 µm. B) Quantification of AF fiber thickness distribution and the NP fibrosis incidence. C) Representative images of 12-month-old discs showing collagen I (COL I) staining, scale bar = 100 µm, n = 3 mice (2m, 1f)/genotype, 2-3 discs/ mouse. Significance for AF collagen area was determined by using an unpaired t-test or Mann-Whitney, as appropriate. Significance for NP fiber area distribution was determined using a χ^2 test. Dotted lines mark the boundaries between the different tissue compartments within the disc.

Supplementary Figure 4

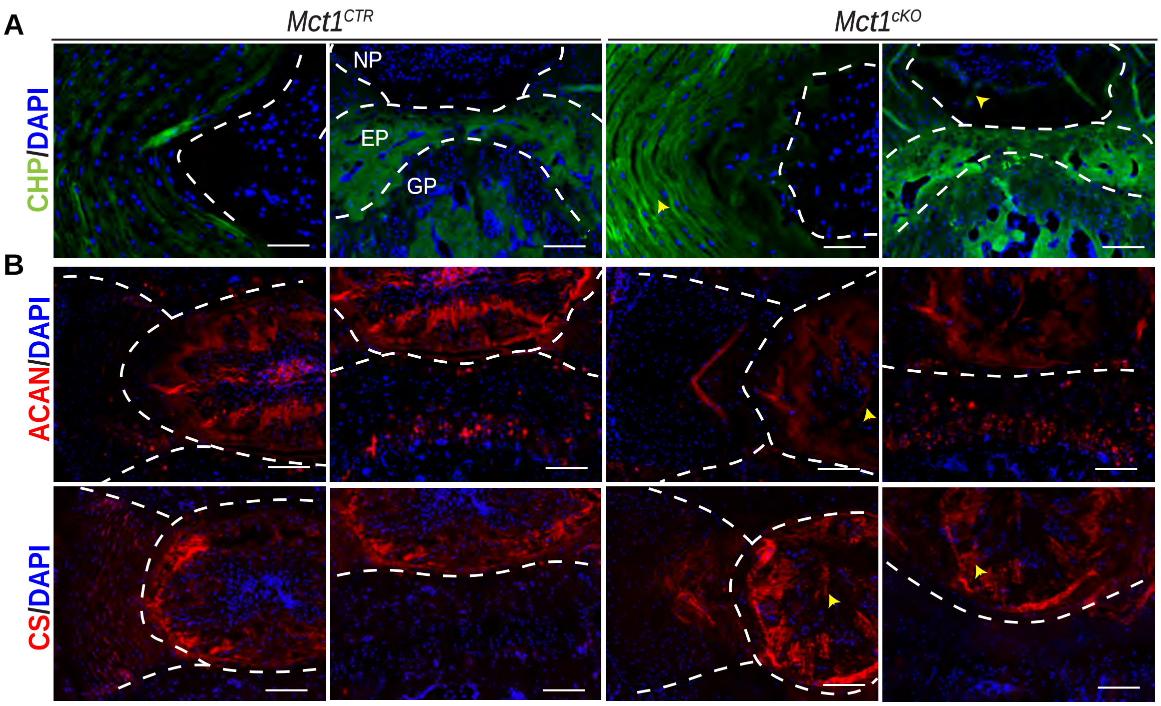

Supplementary Figure 4. **Mct1cKO discs display disorganized matrix.** A-B) Representative images of 12-month-old discs showing collagen hybridizing peptide (CHP), aggrecan (ACAN), chondroitin sulfate (CS) staining, scale bar = 100 µm, n = 3 mice (2m, 1f)/genotype, 2-3 discs/mouse. Yellow arrowheads indicate degenerative changes. Dotted lines mark the boundaries between the different tissue compartments within the disc.

Supplementary Figure 5

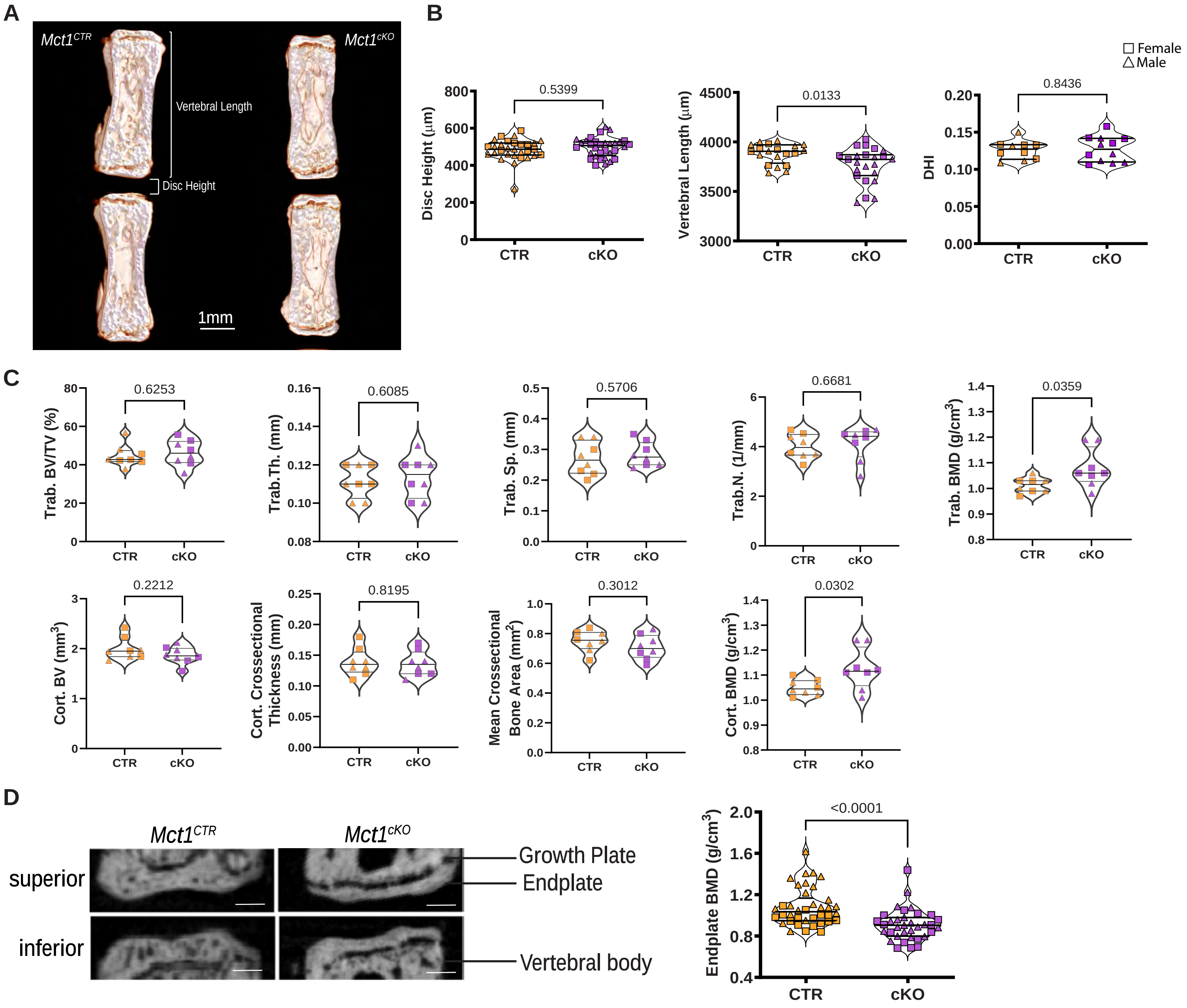

Supplementary Figure 5. **MCT1 loss affects endplate mineralization but does not markedly impact vertebral bone.** A) µCT scan of a caudal vertebral motion segment of 12-month-old $Mct1^{CTR}$ and $Mct1^{cKO}$ mice, scale bar = 1 mm. B) Disc height, vertebral length, and disc height index (DHI). n = 10 mice/genotype (CTR: 6f, 4m; cKO: 5f, 5m), Ca7/8, 8/9/ animal were assessed. C) Trabecular and cortical bone morphology analysis showing bone volume fraction (BV/TV), trabecular thickness (Trab. Th.), separation (Trab. Sp.), number (Trab. N.), and bone mineral density (Trab. BMD), cortical bone volume (BV), cross-sectional thickness, mean cross-sectional bone area, and cortical BMD. n = 4 mice/ genotype (2m, 2f); Ca7-Ca8/mouse were assessed. D) Representative µCT images of superior and inferior endplates of Ca7/8 disc and endplate BMD analysis. n = 10 mice/genotype (CTR: 6f, 4m; cKO 5f, 5m), 2 levels; 4 endplates/mouse were assessed. Scale bar = 125 µm. Data represented as violin plots with median and quartiles. Mann Whitney test or t-test as appropriate was used to test significance.

Supplementary Figure 6

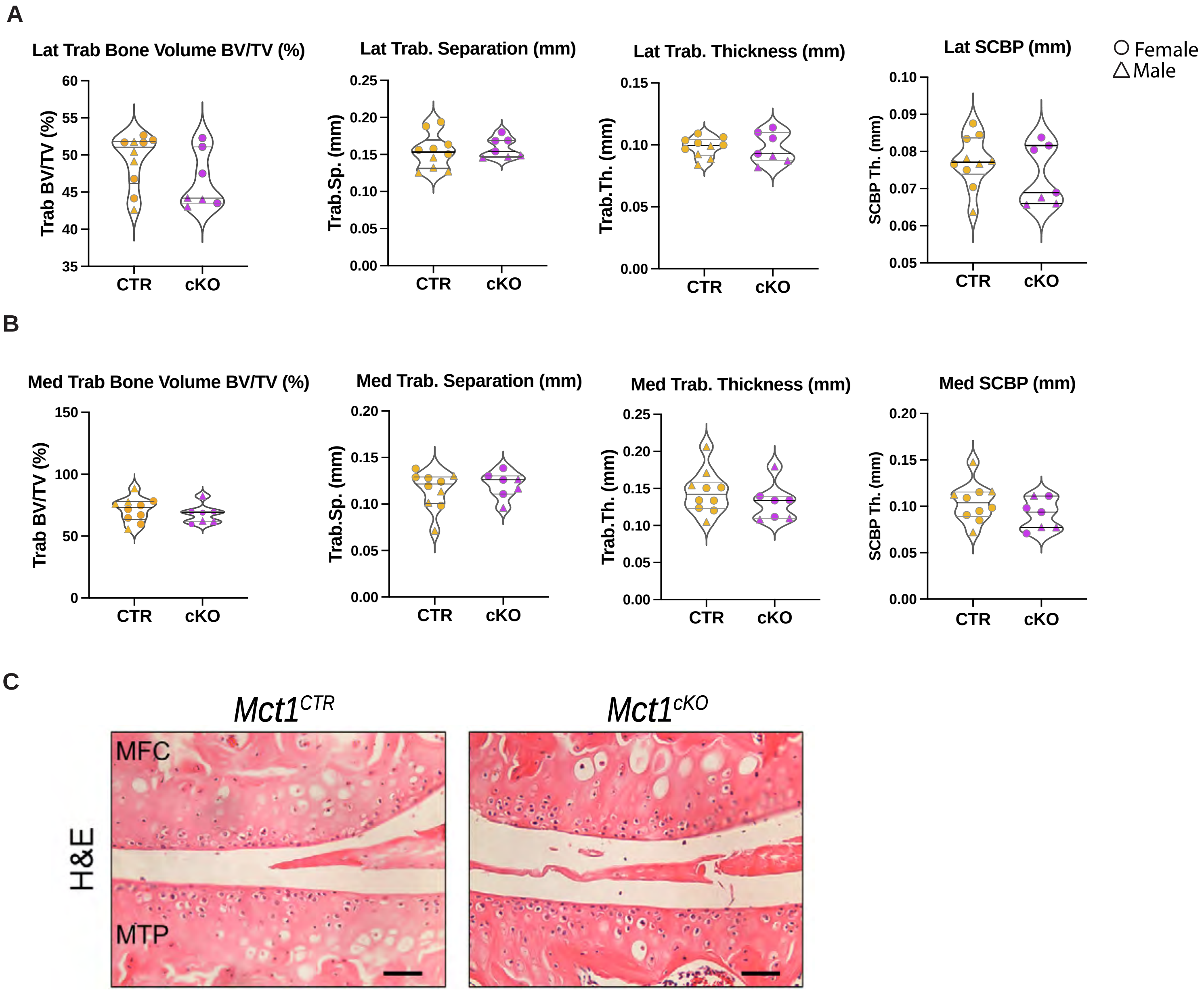

Supplementary Figure 6. **The effect of MCT1 loss on cartilage degradation in mice.** (A-B) Micro-CT analysis of subchondral bone changes (BV/TV, Tb.Sp, Tb.Th, SCBP.Th) on the lateral and medial tibial plateaus of $Mct1^{CTR}$ (CTR) and $Mct1^{cKO}$ (cKO) mice. CTR, n = 10 (6f, 4m); cKO, n = 7 (4f, 3m). Quantitative measurements are shown as violin plots with medians and interquartile ranges. Significance was determined using an unpaired t-test or Mann–Whitney test, as appropriate. C) Representative images of H&E stained midcoronal sections of knee joints from ~1-year-old $Mct1^{CTR}$ and $Mct1^{cKO}$ mice showing no signs of cartilage degradation ($Mct1^{CTR}$ n = 5, $Mct1^{cKO}$ n = 3). MTP, medial tibial plateau; MFC, medial femoral condyle. Scale bar = 50 µm.

Supplementary Figure 7

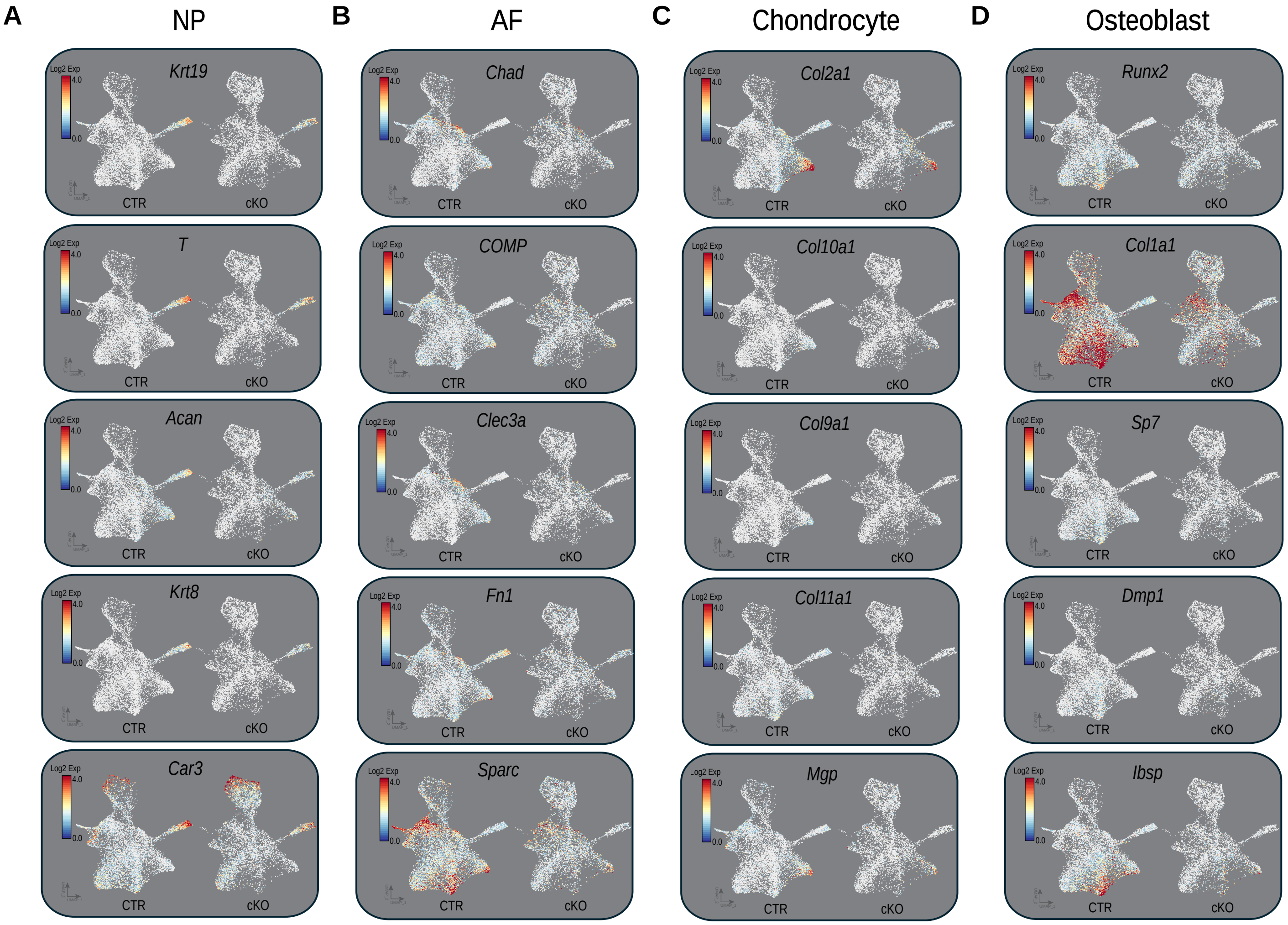

Supplementary Figure 7. **Spatial transcriptomics shows altered gene expression in different disc cell clusters of Mct1^{cKO} mice.** Feature plots showing spatial expression for specific marker genes in (A) NP, (B) AF, (C) chondrocyte, and (D) osteoblast clusters in Mct1^{cKO} and Mct1^{CTR} spinal motion segments, n = 4 mice/genotype; 2m, 2f.

Supplementary Figure 8

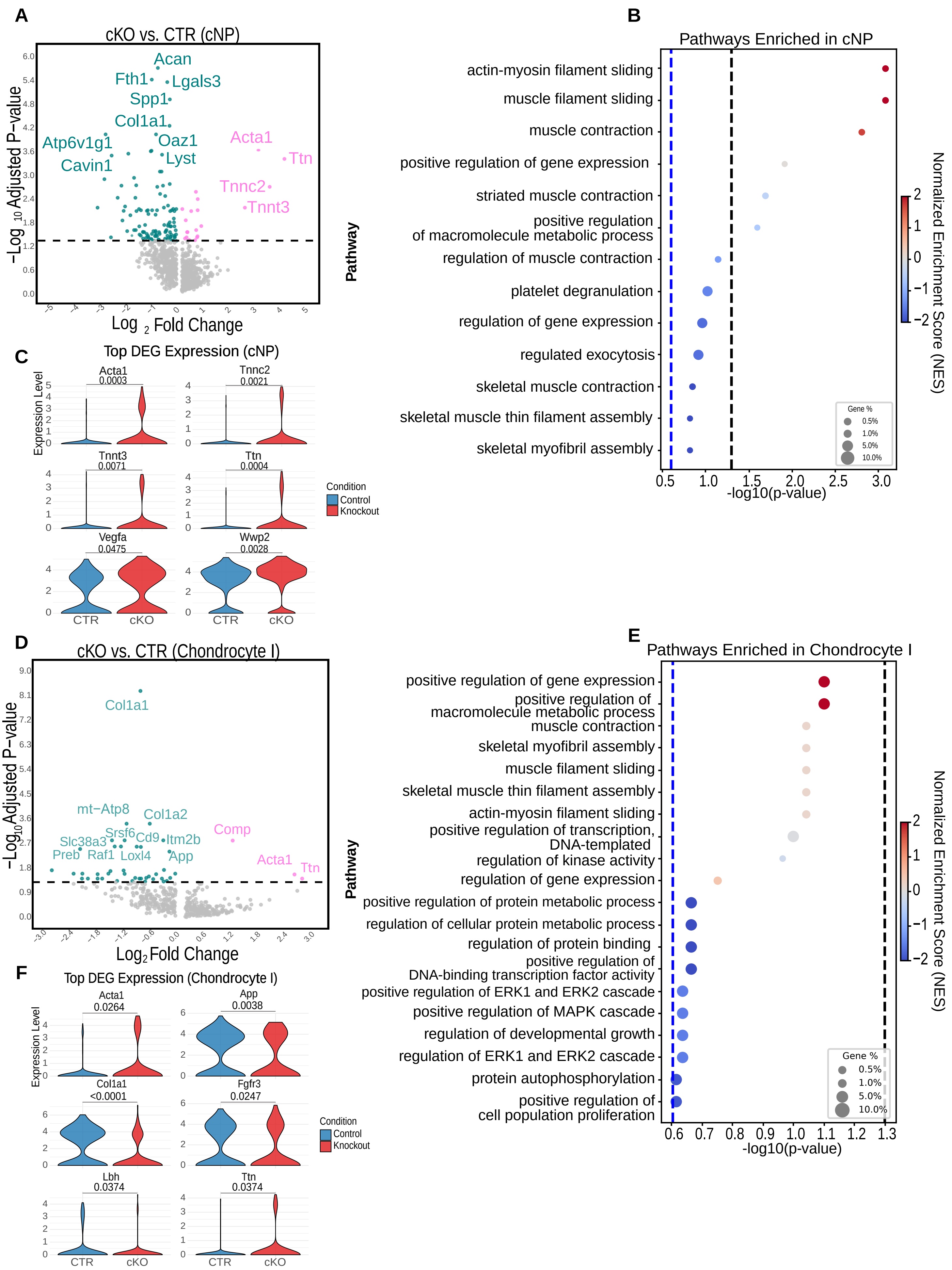

Supplementary Figure 8. **Mct1^{cKO} mice show altered transcriptomic profiles.** A) Volcano plots showing downregulated (green) and upregulated (pink) DEGs in the 'central NP' cluster of Mct1^{cKO}. B) Gene set enrichment analysis (GSEA) of DEGs in 'cNP' cluster from Mct1^{cKO} mice. The blue dotted line represents a q-value of 0.25, while the black dotted line indicates a more stringent q-value of 0.05. C) Violin plots show the distributions of DEGs within the 'cNP' cluster. D) Volcano plots of downregulated- (green) and upregulated (pink) DEGs in the 'Chondrocyte I' cluster from Mct1^{cKO} mice. E) GSEA analysis of DEGs in the 'Chondrocyte I' cluster of Mct1$^{cKO.}$ F) Violin plots showing the distribution of DEGs in the 'Chondrocyte I' cluster of cKO mice (n = 4 mice/genotype; 2m, 2f).

Supplementary Figure 9

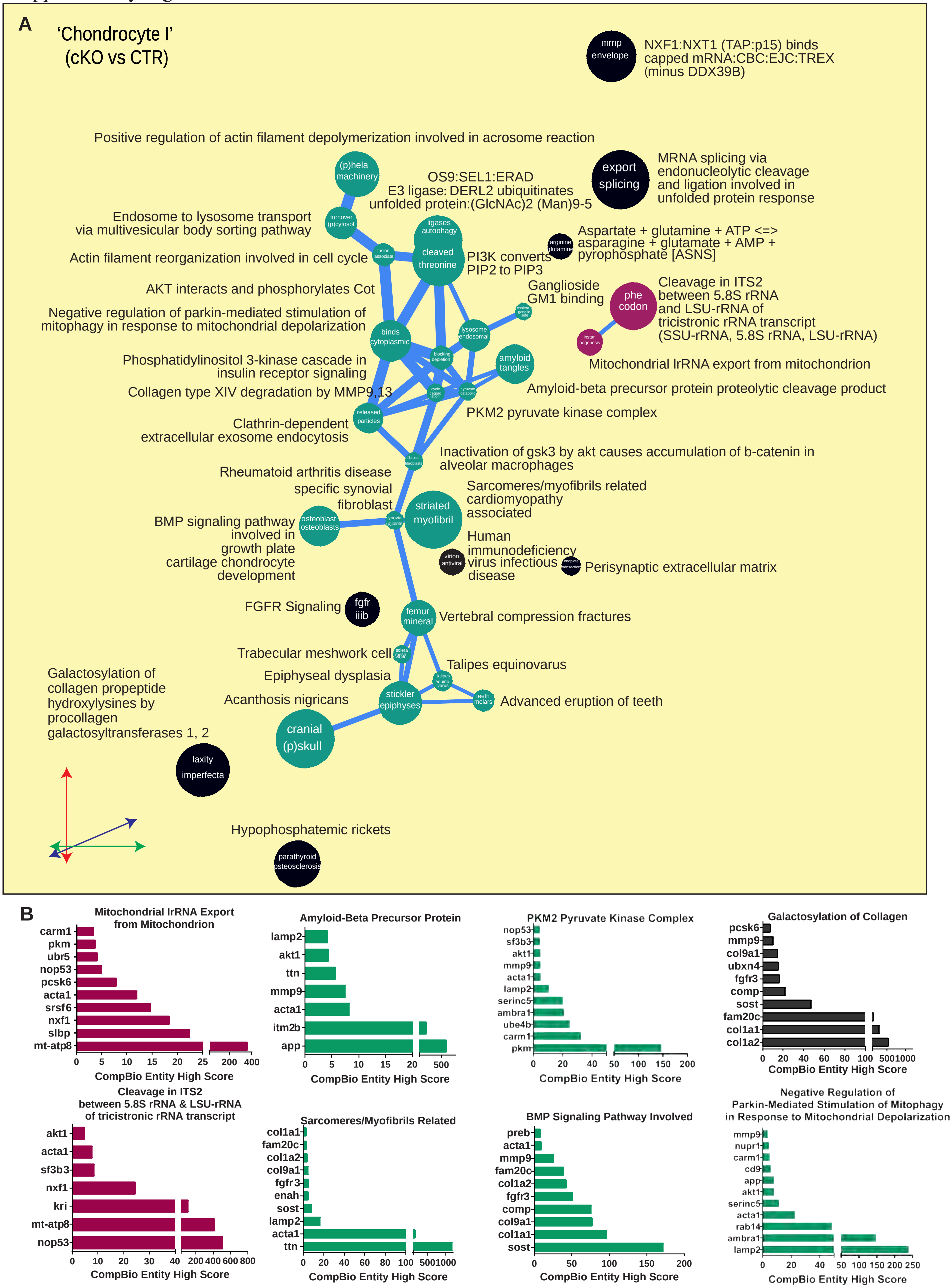

Supplementary Figure 9. M**CT1 deletion disrupts the chondrocyte gene expression.** a) Thematic organization of concepts generated by CompBio, derived from a literature analysis of DEGs in the ‘Chondrocyte I’ cluster in the $Mct1^{cKO}$ mouse discs, highlighting key physiological features impacted by disrupted lactate uptake. Key superclusters of themes include muscle-related pathways, skeletal maturation, actin-cytoskeleton organization and AKT/PI3K/ MAPK signaling (green) and rRNA processing and mitochondrial lrRNA export (red). b) DEGs associated with key themes plotted according to their CompBio entity high score (4 mice/genotype; 2m, 2f).

A

EP Chondrocyte Marker Expression (TPM Values)

	Gene	Samples 1	2	3	4	5	Mean + SD
Chondrogenic Markers	*Acan*	313.2	271.0	294.5	280.4	307.4	293.9 + 17.8
	Col2a1	241.9	230.1	237.0	257.9	264.5	246.3 + 14.4
	Sox9	8.1	6.8	7.3	8.8	7.4	7.7 + 0.8
	Col9a1	69.4	69.9	73.4	71.0	76.1	72.0 + 2.8
	Col11a1	1114.2	933.3	914.9	875.3	887.1	945.0 + 97.3
	Comp	8.6	9.3	10.5	10.0	10.4	9.7 + 0.8
	Tnc	677.7	558.9	608.5	597.5	621.6	612.9 + 13.1
Hypertrophy	*Col10a1*	0.1	0.0	0.0	0.0	0.1	0.0 + 0.0
	Runx2	16.6	13.1	13.1	12.3	12.2	13.5 + 1.8
	Alpl	6.6	5.4	7.1	7.3	7.8	6.8 + 0.9
	Ibsp	3.9	3.6	4.8	5.5	5.2	4.6 + 0.8
	Mmp13	21.1	19.4	19.1	14.8	15.7	18.0 + 2.7
Glucose/Lactate Handling	*Ldha*	2392.7	2355.5	2169.9	2134.7	2118.8	2234.3 + 129.6
	Ldhb	23.0	20.8	23.6	20.7	21.4	21.9 + 1.32
	Slc16a1	68.3	57.9	49.4	50.1	48.4	54.9 + 8.43
	Slc16a3	160.5	161.4	160.9	169.8	189.1	168.4 + 12.23
	Mpc1	168.4	170.7	138.3	134.9	144.2	151.3 + 17.01
	Slc2a1	193.5	188.9	185.1	193.5	189.9	190.2 + 3.51
Endothelial	*Emcn*	0.0	0.0	0.0	0.0	0.0	0 + 0
	Pecam1	ND	ND	ND	ND	ND	ND
Osteoblast	*Bglap*	0.1	1.2	0.0	0.1	0.3	0.3 + 0.47
	Dmp1	0.0	0.0	0.0	0.1	0.1	0.0 + 0.01
Lactate Receptors	*Hcar1*	0.0	0.0	0.0	0.0	0.0	0 + 0
	Gpr132	0.3	0.2	0.2	0.1	0.0	0.2 + 0.11

Supplementary Figure 10. **Marker expression by primary EP cells.** (A) Table showing transcript per million (TPM) values of chondrogenic, hypertrophic, lactate transport, endothelial, osteoblast, and lactate receptor markers in naive EP cells. n = 5 independent isolations

Supplementary Figure 11

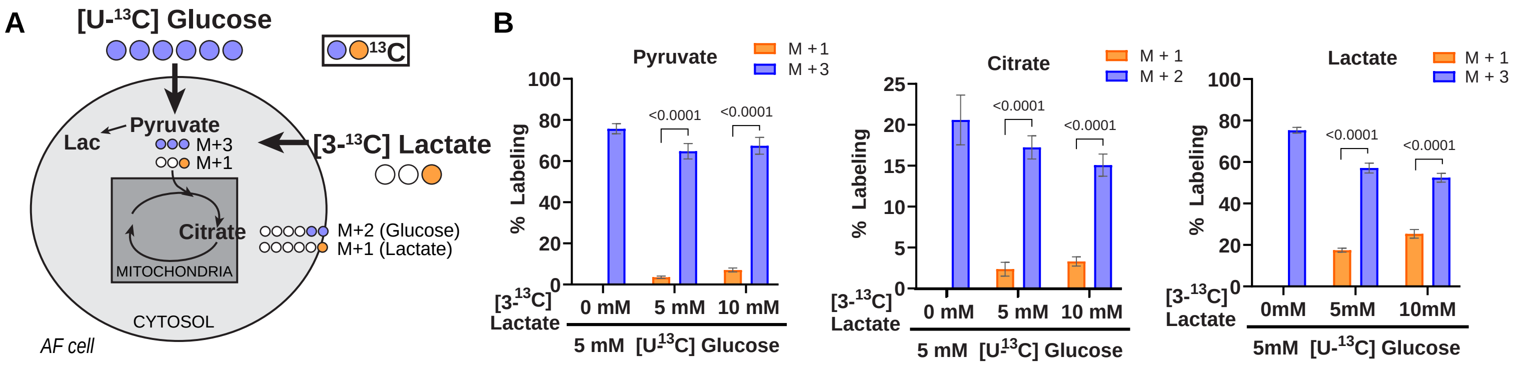

Supplementary Figure 11. **AF cells are primarily glycolytic. Effect of 24-hour lactate supplementation on bioenergetics in AF cells.** A) Schematic representation of dual [U-^{13}C] glucose and [3-^{13}C] lactate tracing, illustrating carbon flow into the TCA cycle. B) Percent labeling of citrate and pyruvate after 24 hr from [U-13C] glucose with increasing concentrations of labeled lactate in AF cells. n = 4 independent samples/condition. Significance was determined using One-way ANOVA with Sidak's post hoc test.

Supplementary Figure 12

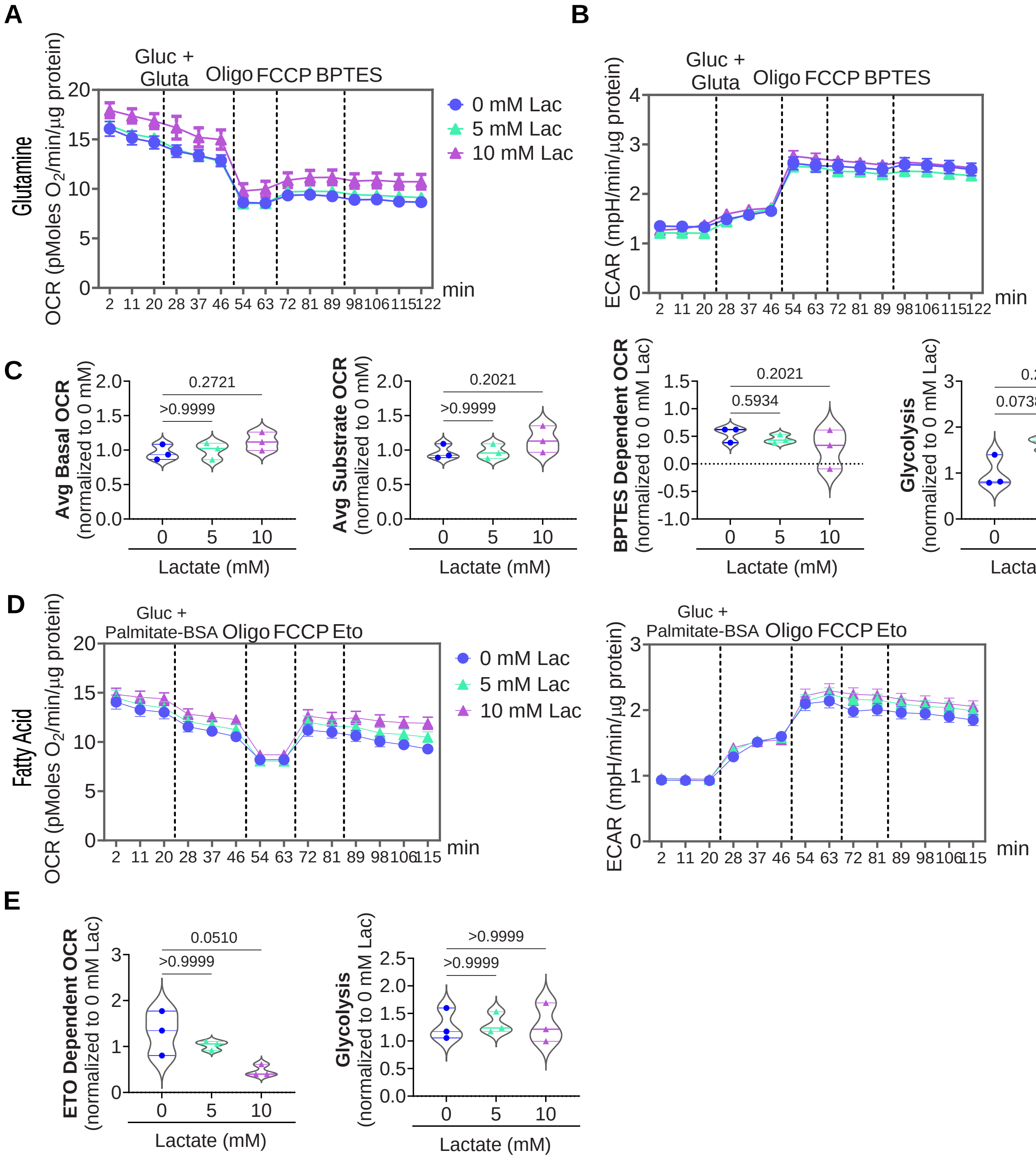

Supplementary Figure 12. **Lactate does not promote OXPHOS switch in EP cells.** Primary EP cells were cultured with sodium L-lactate for one week under osteogenic conditions to measure effect on bioenergetics. (A-B) Normalized OCR and ECAR traces from substrate utilization experiments in a mixed-glucose-glutamine environment, with corresponding (C) average basal and glucose-dependent OCR, BPTES-dependent responses, and glycolysis relative to no-lactate control. D) Normalized ECAR and OCR traces of fatty acid utilization experiment, with corresponding (E) quantification showing etomoxir-dependent OCR and glycolysis parameters. Quantitative measurements are expressed as mean ± SEM (n = 4 experiments, with 4 technical repeats/group/trace) (A, B, D) and as violin plots with medians and interquartile range (C,E). Significance was assessed using Kruskal-Wallis test with Dunn's post hoc analysis (C,E).

Supplementary Figure 13

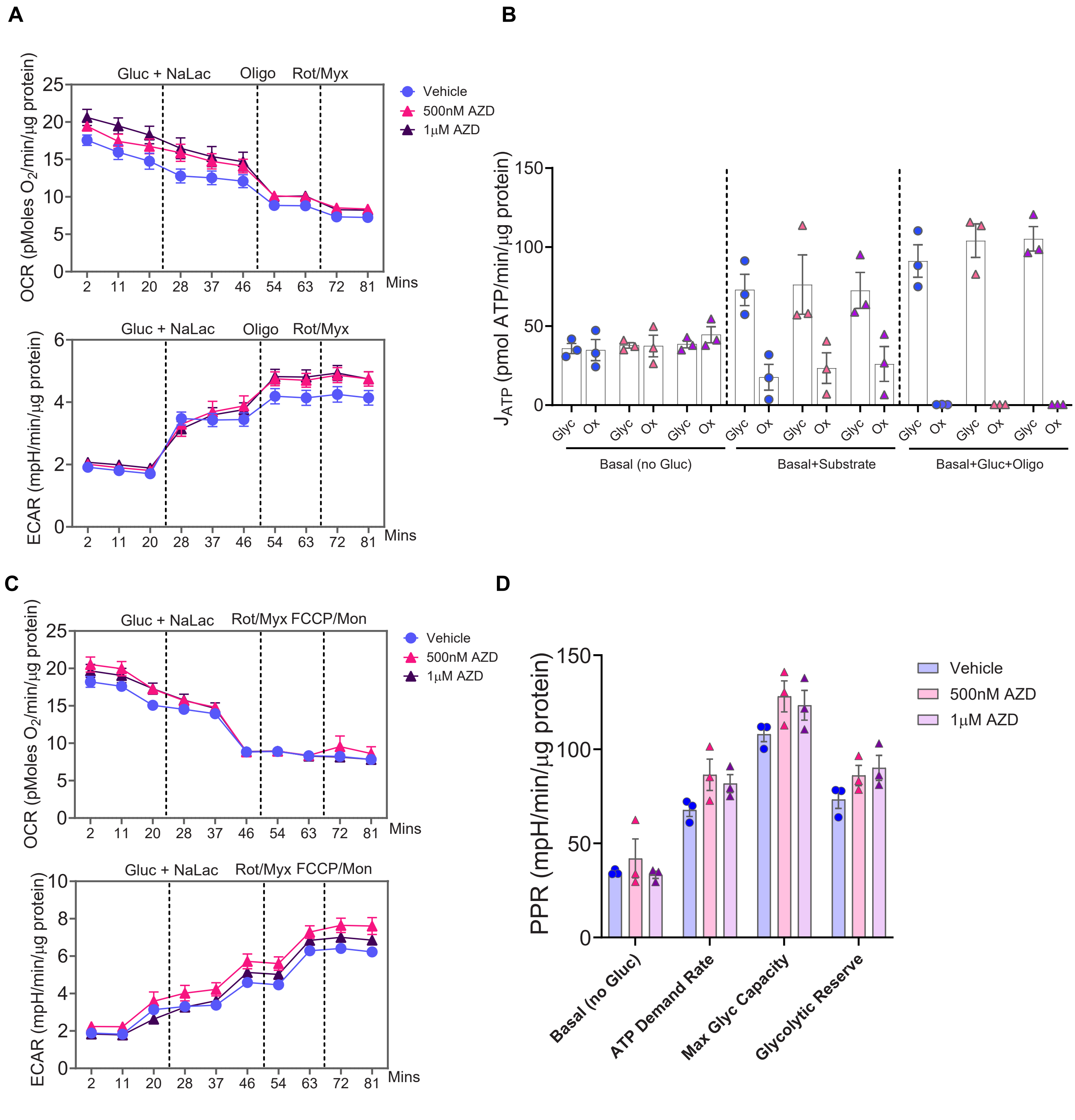

Supplementary Figure 13. **MCT inhibition does not alter AF cell metabolism.** AF cells treated with the MCT1 inhibitor AZD3965 for 24 hours under hypoxic conditions were subjected to a mixed glucose–lactate substrate injection. A) Normalized ECAR and OCR traces, with corresponding B) ATP production rates partitioned into glycolytic and oxidative ATP under basal, substrate, ATP-linked conditions. (n = 3 experiments, with 4 technical repeats/group/trace) (C) Normalized ECAR and OCR traces, with corresponding (D) proton production rates indicating glycolytic capacity and reserve. (n = 3 experiments, with 4 technical repeats/group/trace) Significance was assessed using Kruskal-Wallis test with Dunn's post hoc analysis or one-way ANOVA with Sidak's post hoc test, as appropriate. Data are expressed as mean ± SEM

Supplementary Figure 14

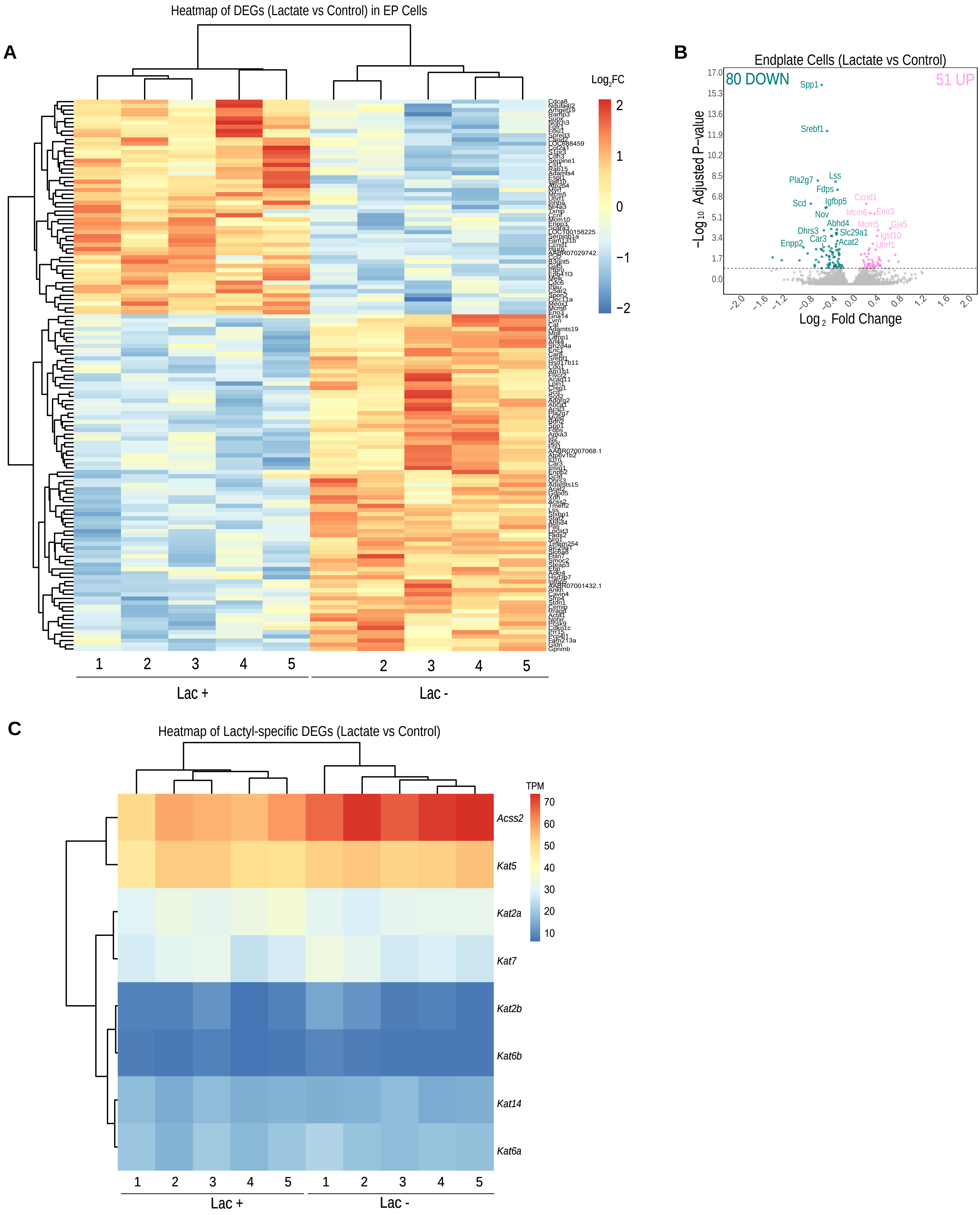

Supplementary Figure 14. **Lactate supplementation induces transcriptional reprogramming in endplate cells.** A) Hierarchical clustering of differentially expressed genes (FDR $p < 0.05$) in lactate-treated endplate cells. B) Volcano plots of down- (green) and up- (pink) regulated DEGs in endplate cells with lactate treatment. C) Hierarchical clustering of curated subset of lactyl-specific DEGs in lactate-treated vs control endplate cells plotted as transcripts per million (TPM). (n = 5 experiments; 2 conditions: lactate or NaCl)

Supplementary Figure 15

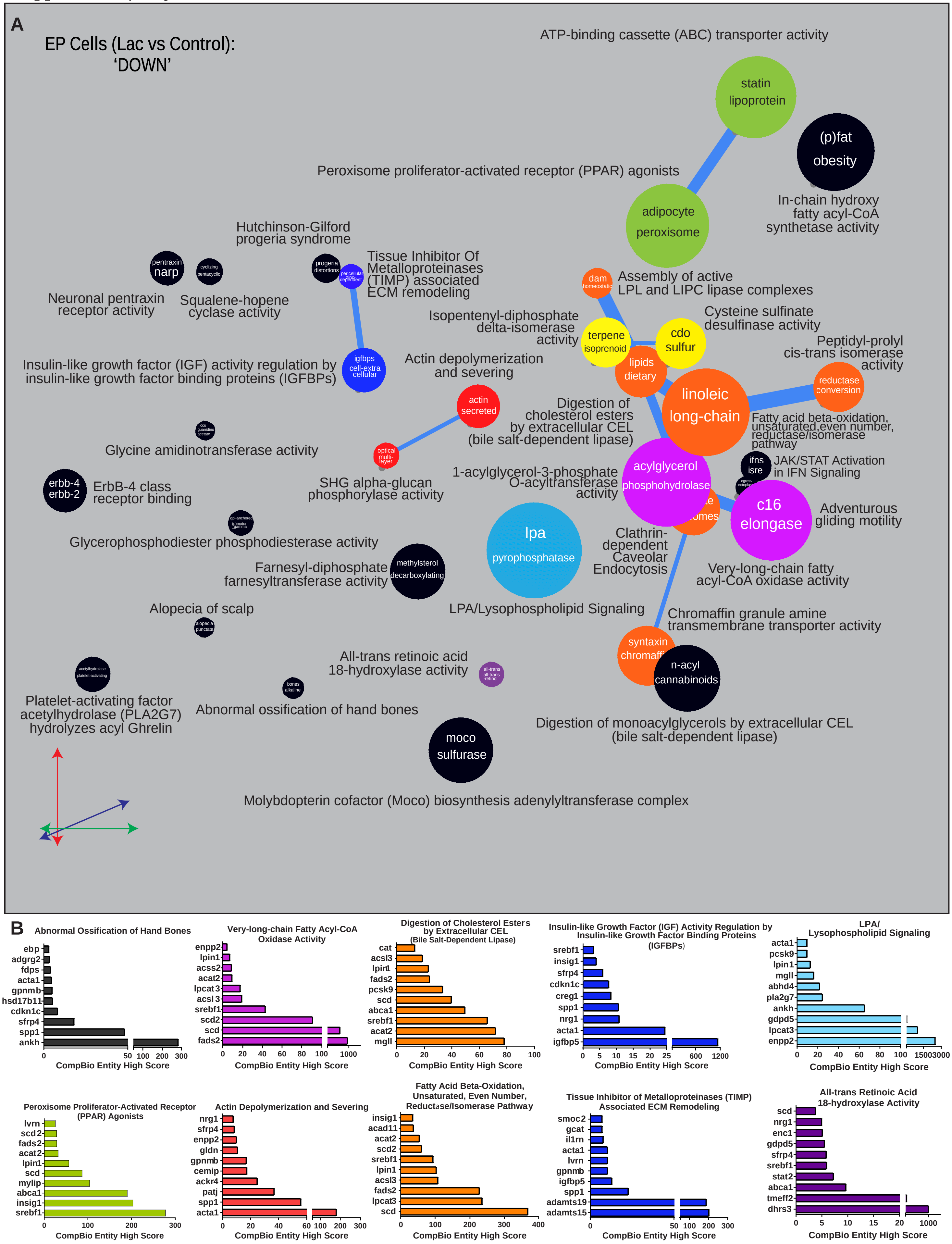

Supplementary Figure 15. **Lactate supplementation suppresses the cartilage sclerotomal phenotype in EP cells.** A) CompBio-generated thematic organization of concepts, derived from literature analysis of differentially downregulated genes in lactate-treated EP cells, reveals key physiological processes affected by lactate. Superclusters of themes associated with these processes include: fatty acids (orange), metabolic enzymes (magenta), calcium-dependent enzyme activities (yellow), ATP-binding cassette transporter activity (green), and growth and tissue remodeling regulation (blue), structural assembly (red). retinoic acid (purple), pyrophosphate (light blue). B) Top DEGs enriched in key themes are displayed as entities based on their CompBio entity high score, (n = 5 experiments; 2 conditions: lactate or NaCl).

Supplementary Figure 16

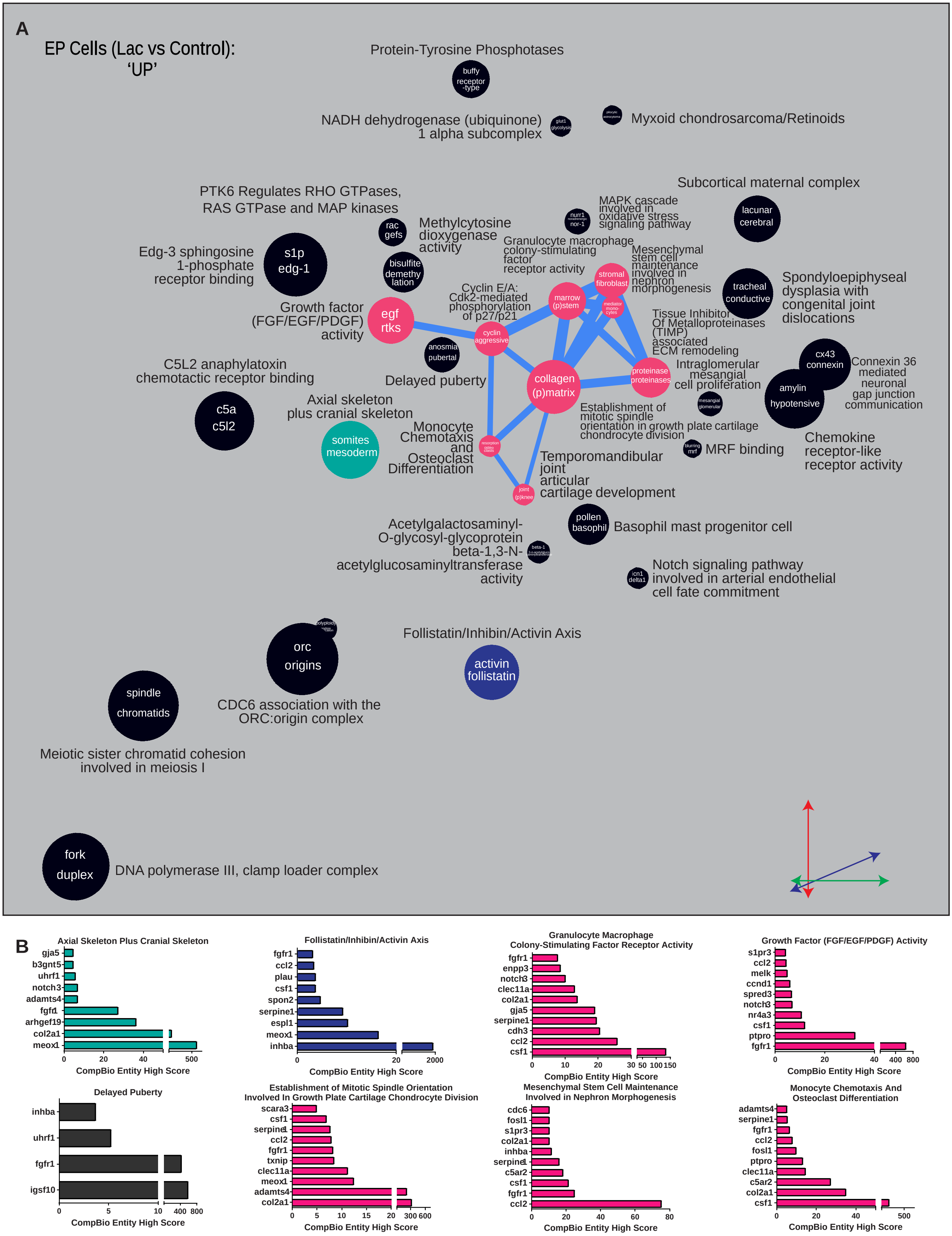

Supplementary Figure 16. **Lactate-treated EP cells upregulate transcriptional programs related to growth regulation and development.** (A) CompBio-generated thematic organization of concepts, derived from literature analysis of differentially upregulated genes in lactate-treated EP cells, reveals key physiological features related to chemotaxis (pink), hormone feedback regulation (blue), and skeletal development (teal) clusters. (B) Top DEGs enriched in key themes are displayed as entities based on their CompBio entity high score, (FDR $p < 0.05$), ($n = 5$ experiments; 2 conditions: lactate or NaCl).

Supplementary Figure 17

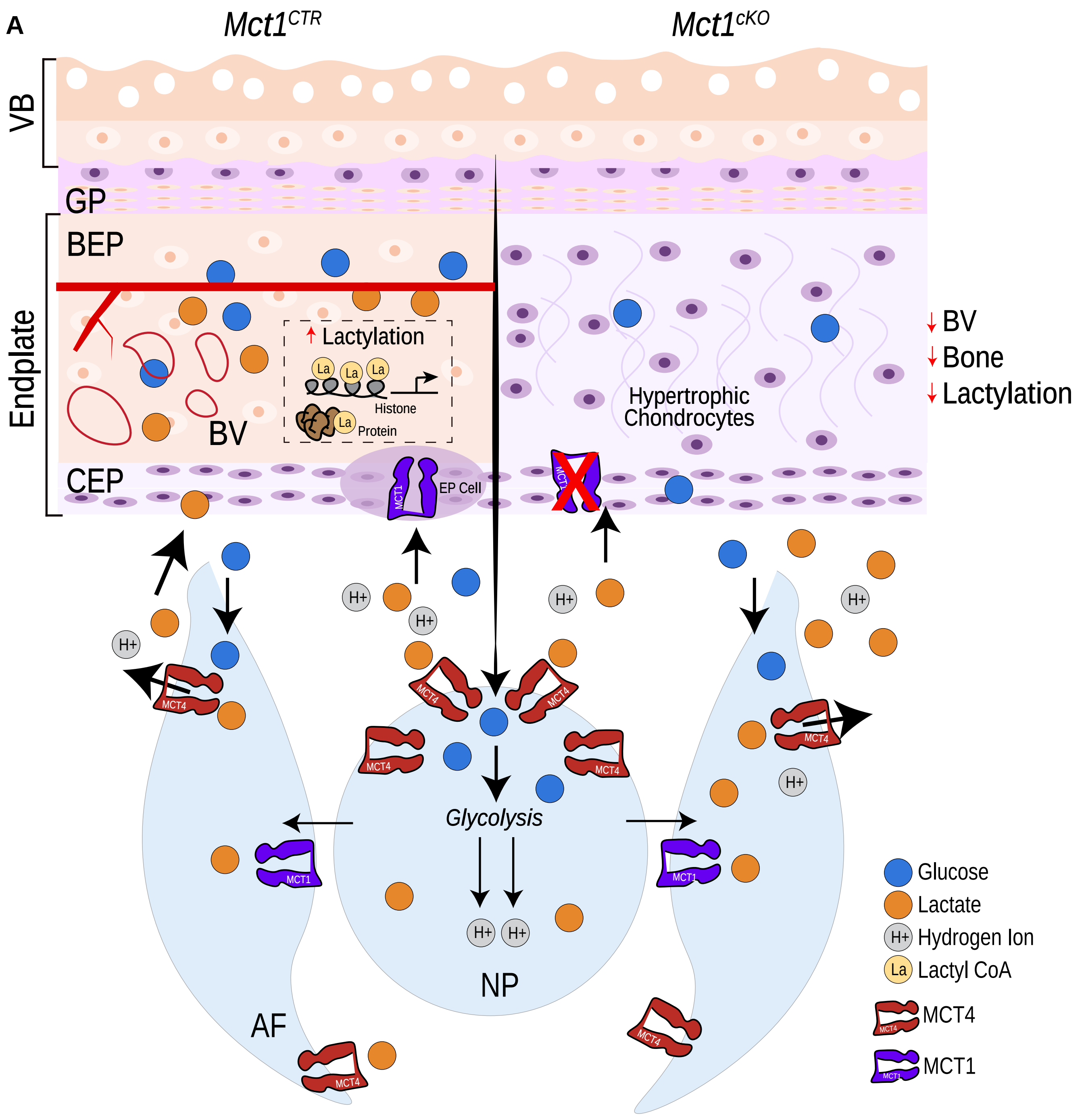

Supplementary Figure 17. **MCT4-MCT1 axis mediates lactate coupling between NP and EP compartments in a growth-dependent manner.** A) Schematic representation of MCT-driven lactate coupling between the nucleus pulposus (NP), annulus fibrosus (AF), and endplate (EP), which lies underneath the growth plate (GP) and consists of the bony endplate (BEP) containing blood vessel (BV) and cartilaginous endplate (CEP), in skeletally mature Mct1^{CTR} and Mct1^{cKO} mice.
