# Supplementary material for: Loss of MCT1 mediated lactate uptake causes delayed endplate maturation and intervertebral disc degeneration
Source: Cell Death Dis. 2026 Jun 3;17(1):698. doi: 10.1038/s41419-026-08942-4 (PMC13448696; doi:10.1038/s41419-026-08942-4)
Supplement: Supplementary file 2 [file 41419_2026_8942_MOESM2_ESM.pdf]

Original Western Blots for Figure 8

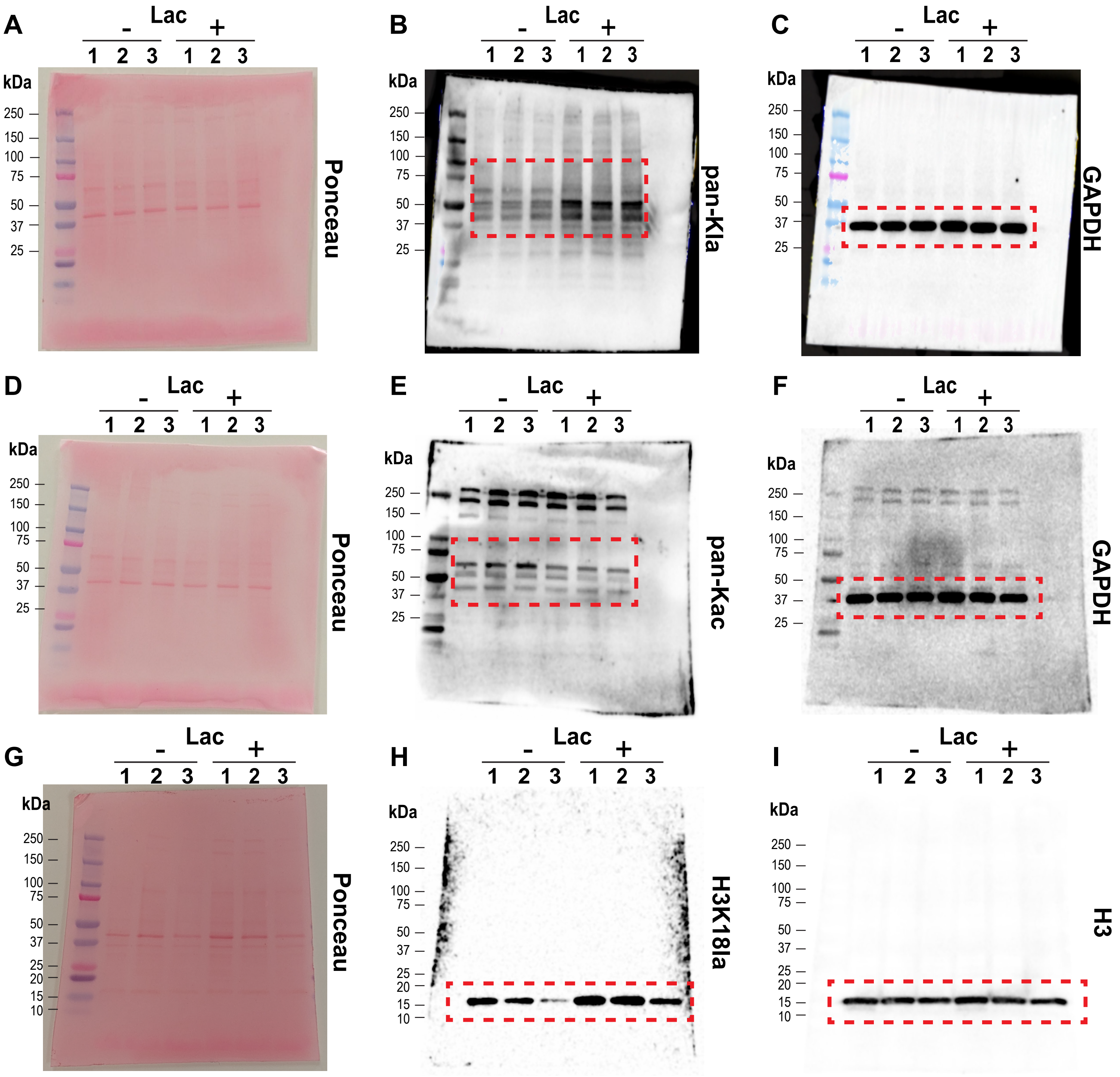

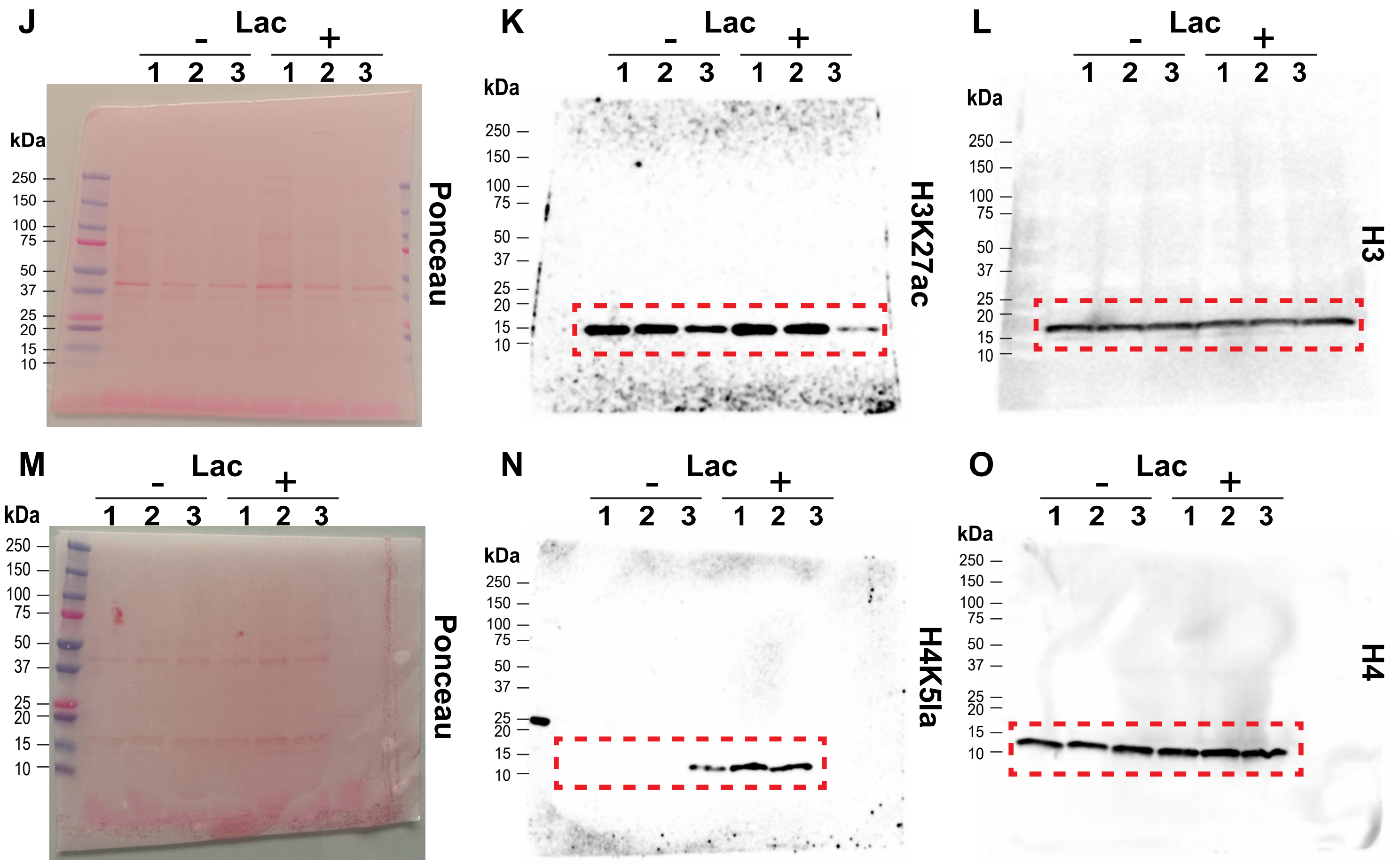

Original Western blot images of lactylation- and acetylation-related markers in EP cells. A) Ponceau Red-stained membrane of pan-lactylation (pan-Kla) blot. B) pan-Kla levels and (C) corresponding GAPDH loading control in EP cells treated with and without lactate. D) Ponceau Red-stained membrane of the pan-acetylation (pan-Kac) blot with (E) pan-Kac levels and (F) corresponding GAPDH levels shown. G) Ponceau Red-stained membrane of H3K18la blot. H) H3K18la levels and (I) corresponding histone H3 levels shown. J) Ponceau Red-stained membrane of H3K27ac blot. K) H3K27ac levels and (L) corresponding H3 levels shown. M) Ponceau Red-stained membrane of H4K5la blot. N) H4K5la levels and (O) corresponding histone H4 levels shown. Pan-lactylation and pan-acetylation blots were performed using whole cell lysate, whereas H3K18la, H3K27ac, and H4K5la blots were performed using purified histones. Blots (B, E, H, K, N) were first probed with the indicated primary antibodies (1:1000), then stripped and re-probed for loading controls (C, F, I, L, O), using antibodies against GAPDH (1:1000), histone H3 (1:3000), or histone H4 (1:1250). Blots shown are representative of three independent experiments.
